# Design, Synthesis and Fungicidal Activity of New 1,2,4-Triazole Derivatives Containing Oxime Ether and Phenoxyl Pyridinyl Moiety

**DOI:** 10.3390/molecules25245852

**Published:** 2020-12-11

**Authors:** Hui Bai, Xuelian Liu, Pengfei Chenzhang, Yumei Xiao, Bin Fu, Zhaohai Qin

**Affiliations:** Department of Chemistry, College of Science, China Agricultural University, Beijing 100193, China; baihui@cau.edu.cn (H.B.); lxl_nuo@126.com (X.L.); chenzhang@cau.edu.cn (P.C.); xiaoymei@cau.edu.cn (Y.X.)

**Keywords:** 1,2,4-triazole, oxime ether, phenoxy pyridinyl, fungicidal activity, CYP51

## Abstract

A series of novel 1,2,4-triazole derivatives containing oxime ether and phenoxy pyridine moiety were designed and synthesized. The new compounds were identified by nuclear magnetic resonance (NMR) spectroscopy and high-resolution mass spectrometry (HRMS). Compound (Z)-1-(6-(4-nitrophenoxy)pyridin-3-yl)-2-(1H-1,2,4-triazol-1-yl)ethan-1-one O-methyl oxime (**5a18**) was further confirmed by X-ray single crystal diffraction. Their antifungal activities were evaluated against eight phytopathogens. The in vitro bioassays indicated that most of the title compounds displayed moderate to high fungicidal activities. Compound (Z)-1-(6-(4-bromo-2-chlorophenoxy)pyridin-3-yl)-2-(1H-1,2,4-triazol-1-yl)ethan-1-one O-methyl oxime (**5a4**) exhibited a broad-spectrum antifungal activities with the EC_50_ values of 1.59, 0.46, 0.27 and 11.39 mg/L against *S. sclerotiorum*, *P. infestans*, *R. solani* and *B. cinerea*, respectively. Compound (Z)-1-(6-(2-chlorophenoxy)pyridin-3-yl)-2-(1H-1,2,4-triazol-1-yl)ethan-1-one O-benzyl oxime (**5b2**) provided the lowest EC_50_ value of 0.12 mg/L against *S. sclerotiorum*, which were comparable to the commercialized difenoconazole. Moreover, homologous modeling and molecular docking disclosed possible binding modes of compounds **5a4** and **5b2** with CYP51. This work provided useful guidance for the discovery of new 1,2,4-triazole fungicides.

## 1. Introduction

The application of agrochemicals in modern agricultural production is the principal measure for ensuring the quantity and quality of crops, vegetables and fruits [1,2]. For all the time using pesticides against various pests, diseases and weeds has been the main research subject of plant protection. Fungicide is an important class of plant protection products which is capable of controlling the growth and reproduction of plant pathogens. Many fungicides with various modes of action have been reported [3,4] and they have become an integral part of efficient food production. However, the frequent use and misuse of fungicides inevitably lead to developing serious resistance [5,6,7]. According to the Fungicide Resistance Action Committee (FRAC), most of the pathogens have already developed medium to high resistance to many existing fungicides. Thus, the search for novel, highly efficient and resistance-overcoming fungicides remains a quite urgent and long-standing task facing agricultural scientists.

Heterocyclic compounds occupied the majority of the commercialized pesticides, play an important role in the development of agricultural fungicides. Among them, the 1,2,4-triazole compounds have become one of the most widely used and studied fungicides due to their low dosage, high selectivity and reduced adverse environmental impact [8,9]. Triazole fungicides have excellent protective and curative power towards a wide spectrum of crop diseases. The biochemical action site of this type of fungicide is lanosterol 14-demethylase (CYP51) dependent on cytochrome P450 in fungal cells [10,11].

**Scheme 1:**
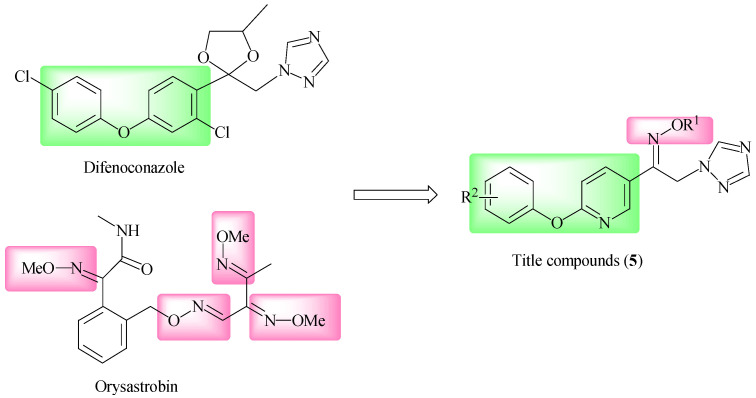
Design of the title compounds.

Pyridine ring is widely present in many medicines and agrochemicals, for example, Picoxystrobin, Boscalid and Haloxyfop and so on. The introduction of pyridine moiety usually reduces the toxicity and improves the selectivity and is considered as an effective strategy in the development of new pesticides [12]. In addition, as a class of versatile bioactive structure oxime ether unit has found its extensive application in many pesticides such as commercialized Orysastrob, Pyribencar, Fenpyroximate and other agricultural activity agents [13,14,15]. In this paper, we selected difenoconazole as a lead compound, the oxime ether moiety and phenoxy pyridinyl group were introduced by the molecular hybridization approach and bioisosterism principle. A series of novel 1, 2, 4-triazole derivatives containing oxime ether and phenoxy pyridine moiety **5a1**~**5a18** and **5b1**~**5b9** were synthesized and evaluated for their antifungal activities (Scheme 1). Moreover, the binding interaction mechanisms of the title compound with SsCYP51 were investigated using molecular docking.

## 2. Results and Discussion

### 2.1. Synthesis

**Scheme 2:**
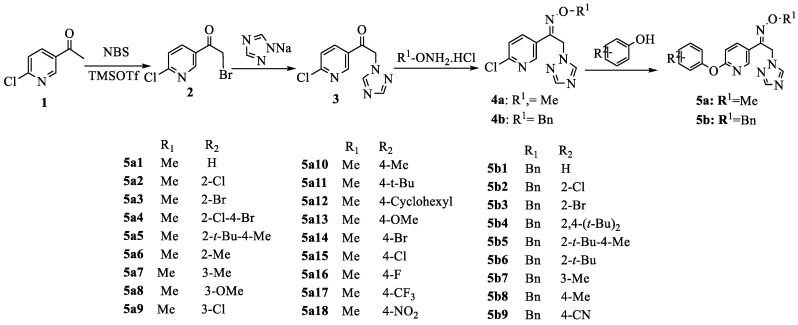
Synthesis of the title compounds.

The synthetic route of the title compounds **5** is illustrated in Scheme 2. The starting material 3-acetyl-6-chloropyridine **1** was prepared according to the method given in the literature [16], Treatment of **1** with NBS (N-Bromosuccinimide) and TMSOTf (Trimethylsilyl trifluoromethanesulfonate)in acetonitrile provided brominated ketone **2 [17]**, which was reacted with sodium triazole to produce 1,2,4-triazole compound **3.** When using liquid brine in the bromination step, a complex mixtures were monitored by TLC (Thin layer chromatography), then replacement of the bromination reagent with NBS/TMSOTf afforded the desired product **2** in good yield. Although there are many approaches for synthesizing 1,2,4-triazole [18,19], the simplest one is the N-alkylation of sodium salt of triazole with halides [20]. Subsequently, the condensation reaction of ketone **3** with O-substituted hydroxylamine hydrochloride led to the key intermediate oxime ether **4**. O-substituted hydroxylamine hydrochloride were commercially available or prepared by the method in the literature. Finally, the title compounds **5** were achieved from the intermediate **4** and different substituted phenol via S_N_2 reaction in DMF (Dimethyl formamide) under basic condition with cesium carbonate as a catalyst [21]. The chemical structures of all of the title compounds **5a1−5a18** and **5b1–5b9** were confirmed by ^1^HNMR and ^13^CNMR spectroscopies and HRMS (High-resolution mass spectrometry) and the physical and chemical properties are provided in the Supplentary materials.

**Figure 1:**
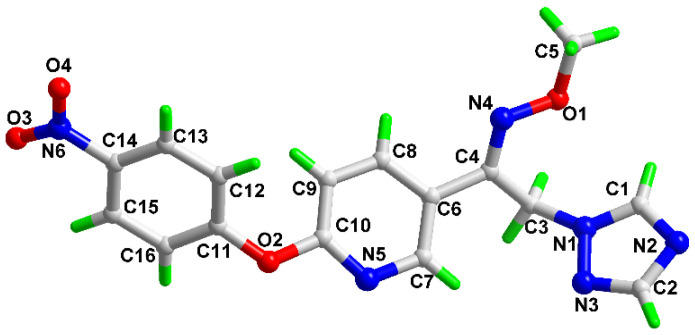
X-ray crystal structure of **5a18** (CCDC2015868).

In order to get a direct insight into the structure of the title compounds, we obtained the single crystal of **5a18** from a mixed solvent of ethyl acetate/methanol and conducted X-ray crystal diffraction analysis. Crystallographic data for compound **5a18** have been deposited in the Cambridge Crystallographic Data Centre (deposition number CCDC-2015868). The perspective view showed the typical “Z” configuration structure as illustrated in Figure 1. As can be seen from the crystallography diagram that the bite angle of N4-C4-C6 is 116.2°, whereas the angle of N4-C4-C3 is 123.9°. This marked difference could be attributed to the larger steric repulsion resulting from the oxime ether and triazole side chain located at same side of C=N bond.

### 2.2. Antifungal Activity

The in vitro fungicidal activities of the title compounds against eight different pathogenic fungi were determined by using mycelium growth rate method and the commercial fungicide difenoconazole was used as the control. The results are summarized in Table 1. In general, methyl oxime ether series **5a** exhibited much higher fungicidal activity than benzyl oxime ether series **5b** (**5a1** vs. **5b1**, **5a2** vs. **5b2**, **5a3** vs. **5b3**, **5a5** vs. **5b5** and **5a10** vs. **5b8**). Comparing with compounds **5a1** to **5a9**, it can be seen that the electron-drawing group at 2-postion of benzene ring was more helpful for the fungicidal activity. Similarly, for benzyl oxime ether series **5b1** to **5b6**, 2-position electron-withdrawing group on the benzene ring was also more conductive to increasing the antifungal activity. It was noteworthy that compounds **5a2**, **5a4**, **5a9**, **5a15** and **5b2** showed much higher inhibitory activities towards fungal pathogens than other compounds. These results could be ascribed to the halogen substituent effect [22,23]. Particularly chlorine atom plays a more important role in improving fungicidal activity of this type of compounds, whatever position of benzene ring it is located at. In addition, the inhibition rates of compounds **5b4**, **5b5** and **5b6** against all the tested fungi were very low, suggesting that for benzyl oxime ether series **5b** a bulky 2-*tert*-butyl group on the benzene ring is not favorable for the fungicidal activity.

According to the preliminary bioassay results, EC_50_ values of ten title compounds against the four tested fungi including *S. sclerotiorum*, *P. infestans*, *R. solani* and *B. cinerea* were determined (Table 2). These compounds generally displayed good to excellent inhibitory activity against *S*. *sclerotiorum* with ranging from 0.12~17.47 mg/L. Especially the EC_50_ values of compounds **5a4** and **5b2** were 1.59 and 0.12 mg/L, respectively, which were comparable to that of commercialized difenoconazole. Moreover, it was observed that compound **5a17** also displayed higher inhibitory activity towards B. cinerea than the control agent (7.48 vs. 8.86 mg/L for EC_50_), which may be due to the effect of -CF_3_ group. It is noted that compound **5a4** with two mixed halogen atom demonstrated **a** broad-spectrum fungicidal activity. The EC_50_ values of compound **5a4** against four fungal pathogens were 1.59, 0.46, 0.27 and 11.39 mg/L, respectively. These findings laid the foundation for further structural optimization and fungicide screening.

### 2.3. Homology Modelling and Molecular Docking

To explore the possible binding modes of the title compounds with CYP51, molecular docking simulation was performed. Because of the absence of the crystal structure of CYP51 from S. sclerotiorum, a homology model was built based on the target-template alignment using ProMod3 (see the detailed procedure in the Supplementary Materials). The sequence alignment of target with template is shown in Figure S1. The constructed homology model of SsCYP51 (PDB code: 6CR2) [24] was used for the subsequent docking study with Glide program (Schrödinger, LLC: New York, NY, USA, 2015) [25,26]. As shown in Figure S2, the modeled structure consisted of 17 helices and 9 beta strands.

**Figure 2:**
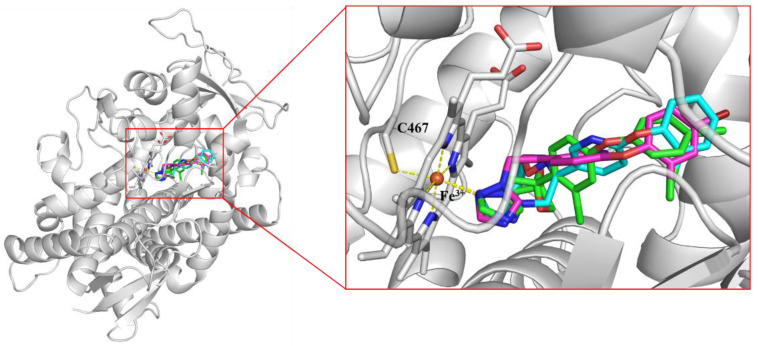
The predicted binding modes of **5a4**, **5b2** and difenoconazole with CYP51. The protein was shown as cartoon, **5a4**, **5b2** and difenoconazole were shown as magenta, cyan and green sticks, respectively.

**Figure 3:**
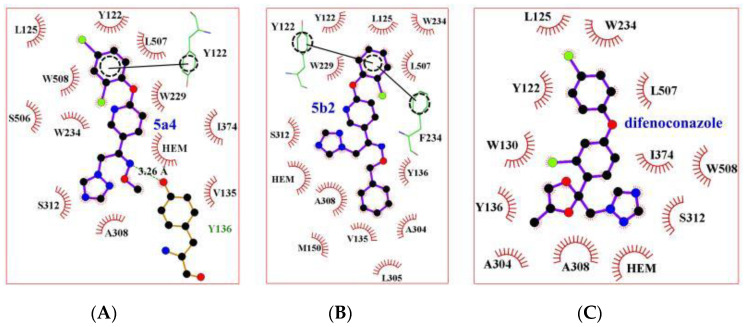
Schematic diagram showed the hydrophobic interactions (shown as starbursts) and H-bond interactions (denoted by dotted green lines) and π-π stacking of three compounds with CYP51. (**A**) Schematic diagram showed the hydrophobic interactions, H-bond interactions and π-π stacking of **5a4** with CYP51; (**B**) Schematic diagram showed the hydrophobic interactions and π-π stacking of **5b2** with CYP51; (**C**) Schematic diagram showed the hydrophobic interactions difenoconazole with CYP51.

Representative compounds **5a4**, **5b2** and the control fungicide difenoconazole were selected to conduct molecular docking simulation with CYP51. As shown in Figure 2, three compounds could be docked into the substrate binding cavity of CYP51 and displayed quite similar binding poses. All the three compounds formed coordinated bonds with Fe^3+^ and established potent hydrophobic interactions with CYP51. The docking score for **5a4**, **5b2** and difenoconazole against CYP51 were −4.787, −5.108 and −6.702, respectively, which was consistent with the order of their EC_50_ values. The detailed interactions (H-bond and hydrophobic interactions) between three compounds with CYP51 were shown in Figure 3A–C. Besides coordinated bonds, H-bond, π-π stacking and hydrophobic interactions are the mainly interactions that maintain these compounds accommodated in the active site of CYP51. In particular, except for having hydrophobic interactions with surrounding residues, **5a4** formed a H-bond through nitrogen atom of oxime ether moiety with residue Y136 (3.26Å) and simultaneously produced π-π stacking through dihalogenated phenyl with an aryl group of Y122. Likewise, **5b2** formed π-π stacking with residues Y122 and F234 apart from establishing hydrophobic interactions with many adjacent residues including Y122, L125, V135, Y136, M150, W229, W234, A304, L305, A308, S312 and L507. The commercial difenoconazole established hydrophobic interactions with surrounding residues Y122, L125, W130, Y136, W234, A304, A308, S312, I374, L507 and W508. Considering the agreement of the docking score order with that of EC_50_ values of three compounds, hydrophobic interactions could play a much more important role in enhancing the fungicidal activity, while the hydrogen bond and π-π stacking interactions seem to be unfavorable for inhibitory activity against *S. scleotiorumin*.

## 3. Materials and Methods

### 3.1. Instruments and Materials

^1^H NMR and ^13^C NMR spectra were obtained using a Bruker Avance DPX300 (or DPX500) spectrometer in CDCl_3_ or DMSO-d6solution with TMS as the internal reference. Chemical shift values (ä) are given in parts per million. Mass spectra were acquired with an Agilent Accurate-Mass-Q-TOF MS 6520 system (Agilent Technologies, Milford, MA, USA) equipped with an electrospray ionization (ESI) source. The single crystal structure analysis was performed using X-ray diffraction with a Bruker D8 Venture X-ray CMOS diffractometer. Melting points were determined with a Cole-Parmer microscope melting point apparatus and are uncorrected.

### 3.2. Synthesis

#### 3.2.1. Synthetic Procedures and Spectral Data for All Title Compounds and Intermediates

The 6-chloro-3-acetylpyridine (**1**) was prepared according to the reported method [16]. 1-(6-chloropyridin-3-yl)ethan-1-one (**1**). white solid; 90% yield, m.p.103–105 °C (Literature value: 104 °C) [27]; ^1^H NMR (500 MHz, CDCl_3_): *ä* 8.93 (d, *J* =1.6 Hz, 1H), 8.26–8.06 (m, 1H), 7.46 (d, *J* = 8.3 Hz, 1H), 2.65 (s, 3H). ^13^C NMR (126 MHz, CDCl_3_): *ä* 195.35, 155.58, 150.12, 138.09, 131.16, 124.49, 26.70.

#### 3.2.2. Synthesis of 2-bromo-1-(6-chloropyridin-3-yl)ethan-1-one (**2**)

The compound **1** (6.2 g, 40 mmol) was dissolved in acetonitrile (100 mL) and cooled to 0 °C. Subsequently, trimethylsilyl trifluoromethanesulfonate (8.9 g, 40 mmol) and N-bromosuccinimide (10.7 g, 60 mmol) were added in batches. The reaction was complete for 6h at room temperature. At the end of the reaction the solvent was evaporated under vacuum, the residue was washed with water and extracted with ethyl acetate. The compound (**2**) was obtained by column chromatography (petroleum ether:ethyl acetate = 5:1, *v*/*v*) purification. white solid; 85% yield, m.p.80–82 °C (Literature value: 78.5–79.5 °C) [28]; ^1^H NMR (500 MHz, CDCl_3_): *δ* 8.98 (d, *J* = 2.4 Hz, 1H), 8.25 (dd, *J* = 8.4, 2.5 Hz, 1H), 7.50 (d, *J* = 8.4 Hz, 1H), 4.43 (s, 2H); ^13^C NMR (126 MHz, CDCl_3_): *δ* 189.21, 156.37, 150.56, 138.85, 128.34, 124.83, 30.03.

#### 3.2.3. Synthesis of 1-(6-chloropyridin-3-yl)-2-(1H-1,2,4-triazol-1-yl)ethan-1-one (**3**)

The intermediate **2** (8.0 g, 34 mmol) was dissolved in acetonitrile (100 mL), triazole sodium (4.7 g, 51 mmol) was added and the mixture was reacted for 4 h. After that, the reaction mixture was concentrated and purified by column chromatography (petroleum ether: ethyl acetate: methanol = 10:10:1) to give a 4.5 g of compound **3**. White solid; 60%yield, m.p.113–116 °C; ^1^H NMR (300 MHz, CDCl_3_): *δ* 9.00 (d, *J* = 2.5 Hz, 1H), 8.29 (s, 1H), 8.23 (d, *J* = 2.5 Hz, 1H), 8.03 (s, 1H), 7.54 (dd, *J* = 8.4, 0.6 Hz, 1H), 5.71 (s, 2H). ^13^C NMR (75 MHz, DMSO): *δ* 191.82, 155.35, 151.85, 150.54, 146.07, 139.48, 129.67, 125.25, 55.89.

#### 3.2.4. Synthesis of (Z)-1-(6-chloropyridin-3-yl)-2-(1H-1,2,4-triazol-1-yl)ethan-1-one O-methyl Oxime (**4a**) and (Z)-1-(6-chloropyridin-3-yl)-2-(1H-1,2,4-triazol-1-yl)ethan-1-one O-benzyl Oxime (**4b**)

Intermediate **3** (2.2 g, 10 mmol), sodium acetate (1.3 g, 15 mmol) and methoxyhydroxylamine hydrochloride (1.0 g, 12 mmol) or benzoxy hydroxylamine hydrochloride (1.9 g, 12 mmol) were dissolved in a mixed solvent (C_2_H_5_OH:H_2_O = 3:1, *V*/*V*) and refluxed for 10 h. The reaction mixture was subsequently cooled to room temperature, inorganic salt was filtered off and the filtrate was concentrated under vacuum. The residue was purified by column chromatography on silica gel (petroleum ether: ethyl acetate:methanol = 10:10:1, *v*/*v*/*v*) to give the corresponding compound **4a** or **4b**. (Z)-1-(6-chloropyridin-3-yl)-2-(1H-1,2,4-triazol-1-yl)ethan-1-one O-methyl oxime (**4a**). white solid; yield 73%, m.p.75–77 °C; ^1^H NMR (300 MHz, CDCl_3_): *δ* 8.77 (d, *J* = 2.5 Hz, 1H), 8.17 (s, 1H), 8.07 (dd, *J* = 8.4, 2.5 Hz, 1H), 7.88 (s, 1H), 7.33 (d, *J* = 8.4 Hz, 1H), 5.36 (s, 2H), 4.09 (s, 3H). ^13^C NMR (75MHz, CDCl_3_): *δ* 152.25, 151.46, 147.89, 147.37, 143.83, 136.17, 128.08, 123.81, 62.93, 42.73.

(Z)-1-(6-chloropyridin-3-yl)-2-(1H-1,2,4-triazol-1-yl)ethan-1-one O-benzyl oxime (**4b**). white solid; 71% yield, m.p.91–93 °C; ^1^H NMR (300 MHz, CDCl_3_): *δ* 8.77 (dd, *J* = 8.0, 2.5 Hz, 1H), 8.13–7.94 (m, 2H), 7.87 (s, 1H), 7.41–7.33 (m, 6H), 5.36 (s, 2H), 5.30 (s, 2H). ^13^C NMR (75 MHz, CDCl_3_): *δ* 152.31, 151.40, 148.31, 147.46, 143.87, 136.24, 135.72, 128.86, 128.38, 128.15, 127.65, 123.84, 77.61, 42.91.

#### 3.2.5. Synthesis of Title Compounds **5a1**–**5a18**, **5b1**–**5b9**

To a 100 mL round-bottom flask, intermediate **4a** (3 mmol) or **4b** (3 mmol), phenol (3.3 mmol) and cesium carbonate (3 mmol) were dissolved in DMF (30 mL). After refluxing for 2 h, a 30 mL of saturated brine solution was added. The solid precipitate was filtered off and the filtrate was separated using a separatory funnel. The water phase was extracted with CH_2_Cl_2_ (3 × 10 mL) and the combined organic phase was dried using anhydrous Na_2_SO_4_. After evaporating the solvent under reduced pressure, the residue was purified by column chromatography (EtOAc:PE = 2:1 *v*/*v*) to obtain pure product.

(Z)-1-(6-phenoxypyridin-3-yl)-2-(1H-1,2,4-triazol-1-yl)ethan-1-one O-methyl oxime (**5a1**) brown oil; 70% yield. ^1^H NMR (300 MHz, CDCl_3_): *δ* 8.59–8.52 (m, 1H), 8.14 (d, *J* = 11.9 Hz, 2H), 7.89 (s, 1H), 7.55–7.37 (m, 2H), 7.29–7.19 (m, 1H), 7.14 (dd, *J* = 8.5, 1.1 Hz, 2H), 6.90 (d, *J* = 0.5 Hz, 1H), 5.35 (s, 2H), 4.07(s, 3H). ^13^C NMR (75MHz, CDCl_3_): *δ* 164.26, 153.28, 151.38, 148.36, 145.80, 137.14, 132.34, 129.38, 124.76, 122.87, 121.01, 110.98, 62.64, 42.93; HRMS calcd. for C_16_H_15_N_5_O_2_ [M+H]^+^ 310.1299, found 310.1293.

(Z)-1-(6-(2-chlorophenoxy)pyridin-3-yl)-2-(1H-1,2,4-triazol-1-yl)ethan-1-one O-methyl oxime (**5a2**) yellow oil; 62% yield. ^1^H NMR (300 MHz, CDCl_3_): *δ* 8.53–8.47 (m, 1H), 8.15 (s, 2H), 7.87 (s, 1H), 7.49–7.42 (m, 1H), 7.29 (dd, *J* = 8.3, 1.6 Hz, 1H), 7.23–7.14 (m, 2H), 6.98 (dd, *J* = 8.7, 0.7 Hz, 1H), 5.33 (s, 2H), 4.05 (s, 3H). ^13^C NMR (75 MHz, CDCl_3_): *δ* 163.44, 151.33, 149.11, 148.32, 145.58, 143.76, 137.32, 130.25, 127.58, 126.98, 126.11, 124.42, 123.59, 110.64, 62.62, 42.92. HRMS calcd for C_16_H_14_ClN_5_O_2_ [M+H]^+^ 344.0909, found 344.0908.

(Z)-1-(6-(2-bromophenoxy)pyridin-3-yl)-2-(1H-1,2,4-triazol-1-yl)ethan-1-one O-methyl oxime (**5a3**) yellow oil; 65% yield. ^1^H NMR (300 MHz, CDCl_3_): *δ* 8.52 (d, *J* = 2.0 Hz, 1H), 8.16 (s, 2H), 7.88 (s, 1H), 7.64 (dd, *J* = 8.0, 1.5 Hz, 1H), 7.35 (dd, *J* = 7.7, 1.2 Hz, 1H), 7.22–7.09 (m, 2H), 6.98 (d, *J* = 8.7 Hz, 1H), 5.34 (s, 2H), 4.06 (s, 3H). ^13^C NMR (75 MHz, CDCl_3_) *δ* 163.43, 151.35, 150.33, 148.32, 145.61, 143.74, 137.32, 133.34, 128.28, 126.41, 124.42, 123.61, 116.28, 110.81, 62.64, 42.94. HRMS calcd. for C_16_H_14_BrN_5_O_2_ [M+H]^+^ 388.0404, found 388.0406.

(Z)-1-(6-(4-bromo-2-chlorophenoxy)pyridin-3-yl)-2-(1H-1,2,4-triazol-1-yl)ethan-1-one O-methyl oxime (**5a4**) yellow solid, 73% yield, m.p.99–102 °C; ^1^H NMR (300 MHz, CDCl_3_): *δ* 8.49 (dd, *J* = 2.5, 0.6 Hz, 1H), 8.15 (dd, *J* = 8.7, 2.5 Hz, 2H), 7.88 (s, 1H), 7.61 (d, *J* = 2.3 Hz, 1H), 7.42 (dd, *J* = 8.6, 2.3 Hz, 1H), 7.08 (d, *J* = 8.6 Hz, 1H), 7.00 (dd, *J* = 8.7, 0.6 Hz, 1H), 5.33 (s, 2H), 4.06 (s, 3H). ^13^C NMR (75 MHz, CDCl_3_) *δ* 163.01, 151.35, 148.39, 148.23, 145.46, 143.76, 137.48, 132.78, 130.66, 128.22, 124.89, 124.73, 118.13, 110.76, 62.67, 42.91. HRMS calcd. for C_16_H_13_BrClN_5_O_2_ [M+H]^+^ 422.0014, found 422.0015.

(Z)-1-(6-(2-(tert-butyl)-4-methylphenoxy)pyridin-3-yl)-2-(1H-1,2,4-triazol-1-yl)ethan-1-one O-methyl oxime (**5a5**) yellow oil; 71% yield. ^1^H NMR (300 MHz, CDCl_3_): *δ* 8.58 (d, *J* = 2.5 Hz, 1H), 8.17 (s, 1H), 8.09 (dd, *J* = 8.7, 2.6 Hz, 1H), 7.90 (d, *J* = 3.5 Hz, 1H), 7.23 (d, *J* = 1.8 Hz, 1H), 7.02 (dd, *J* = 8.4, 1.8 Hz, 1H), 6.86 (dd, *J* = 13.2, 8.4 Hz, 2H), 5.35 (s, 2H), 4.07 (s, 3H), 2.35 (s, 3H), 1.34 (s, 9H). ^13^C NMR (75 MHz, CDCl_3_): *δ* 164.68, 151.35, 149.67, 148.50, 146.06, 143.74, 140.80, 137.01, 134.00, 127.84, 127.21, 123.75, 122.62, 111.02, 62.59, 43.02, 34.20, 30.01, 20.87. HRMS calcd for C_21_H_25_N_5_O_2_ [M+H]^+^ 380.2081, found 380.2080.

(Z)-1-(6-(o-tolyloxy)pyridin-3-yl)-2-(1H-1,2,4-triazol-1-yl)ethan-1-one O-methyl oxime (**5a6**) yellow oil; 75% yield. ^1^H NMR (300 MHz, CDCl_3_): *δ* 8.55 (dd, *J* = 2.5, 0.6 Hz, 1H), 8.21–8.04 (m, 2H), 7.90 (s, 1H), 7.31–7.16 (m, 3H), 7.06 (dd, *J* = 7.8, 1.4 Hz, 1H), 6.94–6.84 (m, 1H), 5.35 (s, 2H), 4.07 (s, 3H), 2.17 (s, 3H).^13^C NMR (101 MHz, CDCl_3_): *δ* 164.56, 151.81, 151.71, 148.78, 146.28, 144.09, 137.49, 131.44, 130.73, 127.16, 125.57, 124.11, 121.87, 110.57, 62.95, 43.31, 16.33. HRMS calcd for C_17_H_17_N_5_O_2_ [M+H]^+^ 324.1455, found 324.1452.

(Z)-1-(6-(m-tolyloxy)pyridin-3-yl)-2-(1H-1,2,4-triazol-1-yl)ethan-1-one O-methyl oxime (**5a7**) browm oil; 70% yield. ^1^H NMR (300 MHz, CDCl_3_): *δ* 8.54 (d, *J* = 2.0 Hz, 1H), 8.16 (s, 2H), 7.89 (s, 1H), 7.53 (d, *J* = 8.6 Hz, 1H), 7.03 (d, *J* = 2.7 Hz, 1H), 6.92 (d, *J* = 8.7 Hz, 1H), 6.86 (s, 1H), 5.35 (s, 2H), 4.07 (s, 3H), 2.39 (s, 3H). ^13^C NMR (75 MHz, CDCl_3_): *δ* 163.89, 152.31, 151.38, 148.31, 145.70, 143.74, 139.14, 137.27, 132.88, 124.36, 123.36, 120.20, 120.10, 111.13, 62.65, 42.91, 22.74. HRMS calcd for C_17_H_17_N_5_O_2_ [M+H]^+^ 324.1455, found 324.1452.

(Z)-1-(6-(3-methoxyphenoxy)pyridin-3-yl)-2-(1H-1,2,4-triazol-1-yl)ethan-1-one O-methyl oxime (**5a8**) white solid, 83% yield, m.p. 100–101.5 °C; ^1^H NMR (300 MHz, CDCl_3_): *δ* 8.56 (d, *J* = 2.5 Hz, 1H), 8.11 (dd, *J* = 15.4, 9.2 Hz, 2H), 7.88 (s, 1H), 7.29 (dd, *J* = 11.2, 5.0 Hz, 1H), 6.90 (d, *J* = 8.7 Hz, 1H), 6.80–6.66 (m, 3H), 5.35 (s, 2H), 4.06 (s, 3H), 3.79 (s, 3H). ^13^C NMR (75 MHz, CDCl_3_): *δ* 163.02, 155.83, 151.39, 150.27, 148.26, 145.55, 143.77, 137.51, 135.92, 124.69, 110.84, 108.38, 107.63, 106.91, 62.69, 56.25, 42.95. HRMS calcd. for C_17_H_17_N_5_O_3_ [M+H]^+^ 340.1404, found 340.1398.

(Z)-1-(6-(3-chlorophenoxy)pyridin-3-yl)-2-(1H-1,2,4-triazol-1-yl)ethan-1-one O-methyl oxime (**5a9**) white solid, 80% yield, m.p. 93–95 °C; ^1^H NMR (300 MHz, CDCl_3_): *δ* 8.56 (d, *J* = 2.1 Hz, 1H), 8.19–8.10 (m, 2H), 7.89 (s, 1H), 7.31 (dd, *J* = 15.5, 7.4 Hz, 1H), 7.23–7.14 (m, 2H), 7.04 (ddd, *J* = 8.1, 2.2, 1.0 Hz, 1H), 6.97–6.91 (m, 1H), 5.35 (s, 2H), 4.07 (s, 3H). ^13^C NMR (75 MHz, CDCl_3_): *δ* 163.58, 153.91, 151.39, 148.27, 145.69, 143.76, 137.36, 134.54, 130.01, 124.89, 124.67, 121.54, 119.27, 111.26, 62.67, 42.90. HRMS calcd. for C_16_H_14_ClN_5_O_2_ [M+H]^+^ 344.0909, found 344.0909.

(Z)-1-(6-(p-tolyloxy)pyridin-3-yl)-2-(1H-1,2,4-triazol-1-yl)ethan-1-one O-methyl oxime (**5a10**) yellow oil; 59% yield. ^1^H NMR (300 MHz, CDCl_3_): *δ* 8.54 (d, *J* = 2.5 Hz, 1H), 8.20–8.03 (m, 2H), 7.89 (s, 1H), 7.20 (d, *J* = 8.4 Hz, 2H), 6.95 (dd, *J* = 37.6, 8.6 Hz, 3H), 5.34 (s, 2H), 4.06 (s, 3H), 2.36 (s, 3H). ^13^C NMR (75 MHz, CDCl_3_): *δ* 164.49, 151.37, 148.45, 145.98, 143.72, 138.49, 137.08, 129.01, 126.73, 125.95, 123.62, 121.30, 119.10, 110.19, 62.60, 42.98, 19.77, 12.15. HRMS calcd. for C_17_H_17_N_5_O_2_ [M+H]^+^ 324.1455, found 324.1451.

(Z)-1-(6-(4-(*t*-butyl)phenoxy)pyridin-3-yl)-2-(1H-1,2,4-triazol-1-yl)ethan-1-one O-methyl oxime (**5a11**) brown oil; 72% yield. ^1^H NMR (300 MHz, CDCl_3_): *δ* 8.57 (d, *J* = 2.2 Hz, 1H), 8.19–8.04 (m, 2H), 7.89 (s, 1H), 7.42 (d, *J* = 8.7 Hz, 2H), 7.06 (d, *J* = 8.7 Hz, 2H), 6.90 (d, *J* = 8.7 Hz, 1H), 5.36 (s, 2H), 4.07 (s, 3H), 1.34 (s, 9H). ^13^C NMR (75 MHz, CDCl_3_): *δ* 164.44, 151.39, 150.85, 148.39, 147.47, 145.82, 143.74, 137.06, 126.27, 123.92, 120.28, 110.92, 62.62, 42.94, 34.11, 31.12. HRMS calcd for C_20_H_23_N_5_O_2_ [M+H]^+^ 366.1924, found 366.1922.

(Z)-1-(6-(4-cyclohexylphenoxy)pyridin-3-yl)-2-(1H-1,2,4-triazol-1-yl)ethan-1-one O-methyl oxime (**5a12**) yellow oil; 61% yield. ^1^H NMR (300 MHz, CDCl_3_): *δ* 8.57 (d, *J* = 2.5 Hz, 1H), 8.20–8.05 (m, 2H), 7.90 (s, 1H), 7.25 (d, *J* = 8.5 Hz, 2H), 7.09–7.02 (m, 2H), 6.90 (d, *J* = 8.7 Hz, 1H), 5.36 (s, 2H), 4.08 (s, 3H), 2.53 (s, 1H), 1.87 (dd, *J* = 8.8, 5.7 Hz, 4H), 1.51–1.16 (m, 6H). ^13^C NMR (101 MHz, CDCl_3_): *δ* 164.82, 151.72, 151.45, 148.73, 146.16, 144.82, 137.40, 128.00, 124.24, 120.97, 111.23, 62.96, 43.98, 43.27, 34.54, 26.90, 26.14. HRMS calcd for C_22_H_25_N_5_O_2_ [M+H]^+^ 392.2081, found 392.2078.

(Z)-1-(6-(4-methoxyphenoxy)pyridin-3-yl)-2-(1H-1,2,4-triazol-1-yl)ethan-1-one O-methyl oxime (**5a13**) brown oil; 68% yield. ^1^H NMR (300 MHz, CDCl_3_): *δ* 8.54 (d, *J* = 2.1 Hz, 1H), 8.18–8.03 (m, 2H), 7.88 (s, 1H), 7.10–7.00 (m, 2H), 6.98–6.84 (m, 3H), 5.34 (s, 2H), 4.06 (s, 3H), 3.81 (s, 3H). ^13^C NMR (75 MHz, CDCl_3_): *δ* 164.71, 156.48, 151.36, 148.39, 146.56, 145.76, 143.73, 137.04, 123.82, 122.05, 114.44, 110.64, 62.60, 55.24, 42.92. HRMS calcd for C_17_H_17_N_5_O_3_ [M+H]^+^ 340.1404, found 340.1402.

(Z)-1-(6-(4-bromophenoxy)pyridin-3-yl)-2-(1H-1,2,4-triazol-1-yl)ethan-1-one O-methyl oxime (**5a14**) yellow oil; 70% yield. ^1^H NMR (300 MHz, CDCl_3_): *δ* 8.54 (d, *J* = 2.5 Hz, 1H), 8.16 (s, 2H), 7.89 (s, 1H), 7.51 (d, *J* = 8.9 Hz, 2H), 7.08–6.99 (m, 2H), 6.94 (d, *J* = 8.7 Hz, 1H), 5.35 (s, 2H), 4.07 (s, 3H). ^13^C NMR (75 MHz, CDCl_3_): *δ* 163.71, 152.29, 151.40, 148.27, 145.63, 143.76, 137.32, 132.34, 124.50, 122.87, 117.62, 111.19, 62.68, 42.90. HRMS calcd for C_16_H_14_BrN_5_O_2_ [M+H]^+^ 388.0403, found 388.0403.

(Z)-1-(6-(4-chlorophenoxy)pyridin-3-yl)-2-(1H-1,2,4-triazol-1-yl)ethan-1-one O-methyl oxime (**5a15**) yellow solid, 65% yield, mp 88–90 °C; ^1^H NMR (400 MHz, CDCl_3_): *δ* 8.52(dd, *J* = 2.5, 0.6 Hz, 1H), 8.16–8.06 (m, 2H), 7.87(s, 1H), 7.34 (d, *J* = 8.9 Hz, 2H), 7.06 (d, *J* = 8.9 Hz, 2H), 6.91 (dd, *J* = 8.7, 0.6 Hz, 1H), 5.33 (s, 2H), 4.05 (s, 3H). ^13^C NMR (101 MHz, CDCl_3_): *δ* 164.14, 152.02, 151.73, 148.59, 145.96, 144.12, 137.65, 130.31, 129.71, 124.78, 122.78, 111.49, 63.02, 43.23.HRMS calcd for C_16_H_14_ClN_5_O_2_ [M+H]^+^ 344.0909, found 344.0912.

(Z)-1-(6-(4-fluorophenoxy)pyridin-3-yl)-2-(1H-1,2,4-triazol-1-yl)ethan-1-one O-methyl oxime (**5a16**) white solid, 77% yield, m.p. 110–112 °C; ^1^H NMR (300 MHz, CDCl_3_): *δ* 8.52 (dd, *J* = 2.5, 0.6 Hz, 1H), 8.11 (dd, *J* = 11.2, 8.7 Hz, 2H), 7.87 (s, 1H), 7.12–7.03 (m, 4H), 6.90 (dd, *J* = 8.7, 0.7 Hz, 1H), 5.34 (s, 2H), 4.05 (s, 3H). ^13^C NMR (75 MHz, CDCl_3_): *δ* 164.11, 161.02, 157.80, 151.35, 149.00, 148.97, 148.32, 145.62, 143.76, 137.23, 124.26, 62.61, 42.88. HRMS calcd for C_16_H_14_FN_5_O_2_ [M+H]^+^ 328.1204, found 328.1201.

(Z)-2-(1H-1,2,4-triazol-1-yl)-1-(6-(4-(trifluoromethyl)phenoxy)pyridin-3-yl)ethan-1-one O-methyl oxime (**5a17**) brown oil; 55% yield. ^1^H NMR (300 MHz, CDCl_3_): *δ* 8.57 (d, *J* = 2.0 Hz, 1H), 8.21–8.12 (m, 2H), 7.90 (s, 1H), 7.67 (d, *J* = 8.5 Hz, 2H), 7.26 (d, *J* = 9.5 Hz, 2H), 7.05–6.96 (m, 1H), 5.37 (s, 2H), 4.09 (s, 3H). ^13^C NMR (101 MHz, CDCl_3_): *δ* 163.62, 156.29, 152.33, 148.56, 145.98, 144.16, 137.85, 127.03, 126.99, 125.30, 122.67, 121.44, 111.94, 63.07, 43.25. HRMS calcd for C_17_H_14_F_3_N_5_O_2_ [M+H]^+^ 378.1172, found 378.1168.

(Z)-1-(6-(4-nitrophenoxy)pyridin-3-yl)-2-(1H-1,2,4-triazol-1-yl)ethan-1-one O-methyl oxime (**5a18**) white solid, 75% yield, m.p. 136–138 °C; ^1^H NMR (300 MHz, CDCl_3_): *δ* 8.57 (d, *J* = 2.0 Hz, 1H), 8.34–8.15 (m, 4H), 7.90 (s, 1H), 7.30 (s, 2H), 7.05 (dd, *J* = 8.6, 0.6 Hz, 1H), 5.38 (s, 2H), 4.09 (s, 3H). ^13^C NMR (75 MHz, CDCl_3_): *δ* 162.60, 158.54, 151.43, 148.13, 145.60, 144.02, 143.82, 137.78, 125.63, 125.14, 120.96, 111.99, 62.77, 42.88. HRMS calcd for C_16_H_14_N_6_O_4_ [M+H]^+^ 355.1149, found 355.1144.

(Z)-1-(6-phenoxypyridin-3-yl)-2-(1H-1,2,4-triazol-1-yl)ethan-1-one O-benzyl oxime (**5b1**) yellow solid, 55% yield, m.p.102–104 °C; ^1^H NMR (300 MHz, CDCl_3_): *δ* 8.63–8.57 (m, 1H), 8.12 (dd, *J* = 9.6, 7.1 Hz, 2H), 7.91 (s, 1H), 7.47–7.35 (m, 7H), 7.25 (s, 1H), 7.17 (dd, *J* = 8.5, 1.1 Hz, 2H), 6.94 (d, *J* = 8.7 Hz, 1H), 5.38 (s, 2H), 5.30 (s, 2H). ^13^C NMR (75 MHz, CDCl_3_): *δ* 164.29, 153.31, 151.31, 148.78, 145.90, 145.75, 143.77, 137.19, 136.09, 129.38, 128.34, 128.20, 124.76, 124.19, 121.03, 110.97, 43.09. HRMS calcd for C_22_H_19_N_5_O_2_ [M+H]^+^ 386.1611, found 386.1609.

(Z)-1-(6-(2-chlorophenoxy)pyridin-3-yl)-2-(1H-1,2,4-triazol-1-yl)ethan-1-one O-benzyl oxime (**5b2**) yellow oil; 72% yield. ^1^H NMR (300 MHz, CDCl_3_): *δ* 8.54 (dd, *J* = 2.5, 0.6 Hz, 1H), 8.15 (dd, *J* = 8.7, 2.5 Hz, 1H), 8.07 (s, 1H), 7.88 (s, 1H), 7.45 (d, *J* = 1.5 Hz, 1H), 7.37 (d, *J* = 0.8 Hz, 6H), 7.21 (dd, *J* = 4.8, 3.8 Hz, 2H), 6.99 (dd, *J* = 8.7, 0.6 Hz, 1H), 5.33 (s, 2H), 5.27 (s, 2H). ^13^C NMR (75 MHz, CDCl_3_): *δ* 163.48, 151.30, 149.14, 148.76, 145.71, 143.82, 137.39, 136.12, 130.28, 128.33, 128.20, 127.60, 127.01, 126.14, 124.51, 123.64, 110.64, 77.28, 43.09. HRMS calcd. for C_22_H_18_ClN_5_O_2_ [M+H]^+^ 420.1221, found 420.1223.

(Z)-1-(6-(2-bromophenoxy)pyridin-3-yl)-2-(1H-1,2,4-triazol-1-yl)ethan-1-one O-benzyl oxime (**5b3**) yellow oil; 75% yield. ^1^H NMR (300 MHz, CDCl_3_): *δ* 8.60–8.49 (m, 1H), 8.16 (dd, *J* = 8.7, 2.5 Hz, 1H), 8.07 (s, 1H), 7.88 (s, 1H), 7.65 (dd, *J* = 8.0, 1.5 Hz, 1H), 7.45–7.31 (m, 6H), 7.23–7.09 (m, 2H), 7.00 (d, *J* = 8.7 Hz, 1H), 5.34 (s, 2H), 5.27 (s, 2H). ^13^C NMR (75 MHz, CDCl_3_): *δ* 163.46, 151.31, 150.34, 148.74, 145.73, 143.79, 137.38, 136.10, 133.35, 128.33, 128.20, 126.44, 124.49, 123.65, 116.31, 110.80, 77.25, 43.11. HRMS calcd for C_22_H_18_BrN_5_O_2_ [M+H]^+^ 464.0716, found 464.0721.

(Z)-1-(6-(2,4-di-*tert*-butylphenoxy)pyridin-3-yl)-2-(1H-1,2,4-triazol-1-yl)ethan-1-one O-benzyl oxime (**5b4**) yellow oil; 70% yield. ^1^H NMR (300 MHz, CDCl_3_): *δ* 8.66 (dd, *J* = 2.5, 0.6 Hz, 1H), 8.11 (s, 2H), 7.91 (s, 1H), 7.52–7.47 (m, 1H), 7.40 (s, 5H), 7.30–7.22 (m, 1H), 6.91 (d, *J* = 8.3 Hz, 2H), 5.38 (s, 2H), 5.30 (s, 2H), 1.39 (d, *J* = 8.2 Hz, 18H). ^13^C NMR (75 MHz, CDCl_3_): *δ* 164.60, 151.29, 149.59, 148.86, 146.95, 146.19, 143.79, 140.09, 137.07, 136.12, 128.34, 128.19, 124.07, 123.85, 123.59, 121.91, 111.10, 43.16, 34.52, 34.32 31.22, 30.06. HRMS calcd. for C_30_H_35_N_5_O_2_ [M+H]^+^ 498.2864, found 498.2863.

(Z)-1-(6-(2-(*tert*-butyl)-4-methylphenoxy)pyridin-3-yl)-2-(1H-1,2,4-triazol-1-yl)ethan-1-one O-benzyl oxime (**5b5**) yellow oil; 61% yield. ^1^H NMR (300 MHz, CDCl_3_): *δ* 8.61 (dd, *J* = 2.5, 0.6 Hz, 1H), 8.08 (s, 2H), 7.89 (s, 1H), 7.38 (d, *J* = 0.7 Hz, 5H), 7.24 (d, *J* = 1.9 Hz, 1H), 7.07–6.99 (m, 1H), 6.93–6.82 (m, 2H), 5.36 (s, 2H), 5.28 (s, 2H), 2.36 (s, 3H), 1.35 (s, 9H). ^13^C NMR (75 MHz, CDCl_3_): *δ* 164.71, 151.27, 149.67, 148.87, 146.17, 143.78, 140.82, 137.06, 136.12, 134.02, 128.35, 128.33, 128.19, 127.85, 127.24, 123.82, 122.66, 111.02, 43.17, 34.21, 30.04, 29.87, 20.89. HRMS calcd for C_27_H_29_N_5_O_2_ [M+H]^+^ 456.2394, found 456.2394.

(Z)-1-(6-(2-(tert-butyl)phenoxy)pyridin-3-yl)-2-(1H-1,2,4-triazol-1-yl)ethan-1-one O-benzyl oxime (**5b6**) yellow oil; 65% yield. ^1^H NMR (300 MHz, CDCl_3_): *δ* 8.60 (d, *J* = 2.1 Hz, 1H), 8.18–8.07 (m, 2H), 7.89 (s, 1H), 7.55 (d, *J* = 2.4 Hz, 1H), 7.38 (d, *J* = 0.6 Hz, 6H), 6.99–6.82 (m, 2H), 5.36 (s, 2H), 5.28 (s, 2H), 1.36 (d, *J* = 6.5 Hz, 9H). ^13^C NMR (75 MHz, CDCl_3_): *δ* 164.26, 151.68, 151.40, 149.07, 146.42, 144.16, 143.88, 137.70, 136.38, 130.72, 129.93, 128.71, 128.70, 128.58, 124.90, 124.76, 118.11, 111.65, 77.63, 43.48, 34.91, 30.09. HRMS calcd. for C_26_H_27_N_5_O_2_ [M+H]^+^ 442.2237, found 442.2237.

(Z)-1-(6-(*m*-tolyloxy)pyridin-3-yl)-2-(1H-1,2,4-triazol-1-yl)ethan-1-one O-benzyl oxime (**5b7**) white solid, 85% yield, 97–99 °C; ^1^H NMR (300 MHz, CDCl_3_): *δ* 8.56 (d, *J* = 2.5 Hz, 1H), 8.17–8.04 (m, 2H), 7.88 (s, 1H), 7.54 (d, *J* = 8.6 Hz, 1H), 7.38 (s, 5H), 7.03 (d, *J* = 2.7 Hz, 1H), 6.93 (d, *J* = 8.7 Hz, 1H), 6.87 (s, 1H), 5.36 (s, 2H), 5.28 (s, 2H), 2.40 (s, 3H). ^13^C NMR (75 MHz, CDCl_3_): *δ* 163.94, 152.31, 151.33, 148.71, 145.81, 143.77, 139.16, 137.31, 136.05, 132.91, 128.34, 128.22, 124.42, 123.39, 120.25, 120.13, 111.11, 77.28, 43.09, 22.76. HRMS calcd for C_23_H_21_N_5_O_2_ [M+H]^+^ 400.1768, found 400.1767.

(Z)-4-((5-(1-((benzyloxy)imino)-2-(1H-1,2,4-triazol-1-yl)ethyl)pyridin-2-yl)oxy)benzonitrile (**5b8**) yellow solid, 74% yield, 116–118 °C; ^1^H NMR (300 MHz, CDCl_3_): *δ* 8.59 (dd, *J* = 2.5, 0.7 Hz, 1H), 8.21 (dd, *J* = 8.7, 2.5 Hz, 1H), 8.11 (s, 1H), 7.90 (s, 1H), 7.75–7.67 (m, 2H), 7.43–7.36 (m, 5H), 7.27 (d, *J* = 8.9 Hz, 2H), 7.04 (dd, *J* = 8.7, 0.7 Hz, 1H), 5.39 (s, 2H), 5.30 (s, 2H). ^13^C NMR (75 MHz, CDCl_3_): *δ* 162.74, 156.93, 151.35, 148.58, 145.68, 143.87, 139.22, 137.76, 136.56, 135.98, 135.27, 133.52, 128.35, 128.32, 128.25, 125.49, 121.53, 118.18, 111.86, 108.00, 43.06, 29.28. HRMS calcd for C_23_H_18_N_6_O_2_ [M+H]^+^ 411.1564, found 411.1564.

(Z)-1-(6-(p-tolyloxy)pyridin-3-yl)-2-(1H-1,2,4-triazol-1-yl)ethan-1-one O-benzyl oxime (**5b9**) yellow solid, 71% yield, 108–110 °C; ^1^H NMR (300 MHz, CDCl_3_): *δ* 8.60–8.52 (m, 1H), 8.06 (s, 2H), 7.88 (s, 1H), 7.38 (d, *J* = 0.6 Hz, 5H), 7.20 (s, 2H), 7.03 (d, *J* = 8.5 Hz, 2H), 6.89 (s, 1H), 5.35 (s, 2H), 5.27 (s, 2H), 2.37 (s, 3H). ^13^C NMR (75 MHz, CDCl_3_): *δ* 164.57, 151.30, 150.96, 148.83, 145.91, 143.76, 137.10, 136.13, 134.39, 129.92, 128.33, 128.19, 123.97, 120.88, 110.80, 43.09, 20.54. HRMS calcd. for C_23_H_21_N_5_O_2_ [M+H]^+^ 400.1768, found 400.1767.

### 3.3. Fungicidal Activity Assay

The in vitro fungicidal activities against plant pathogens were tested according to the reported method [29]. The medium was amended with aliquots of each tested compound solution to provide a concentration of 50 mg/L. The tested compounds were dissolved in 0.3 mL of dimethyl sulfoxide (DMSO) and added aseptically to molten agar after autoclaving, when the agar had cooled to 45−50 °C. The concentration of solvent never exceeded 0.1 mg/L. The mixed medium without sample was used as the blank control. The inocula, 5 mm in diameter, were removed from the margins of actively growing colonies of mycelium, placed in the centers of the above plates. Three replicates were done for each concentration and the control plates were sealed with parafilm and incubated at 26 °C in darkness. The diameter of the mycelium was measured after several days. The inhibition percent was used to describe the control efficiency of the compounds. Inhibition percent (%) = (hyphal diameter in the control—hyphal diameter in the treatment)/hyphal diameter in the control. The results are summarized in Table 1.

A 20 mg/mL stock solution was diluted with PDA to obtain a series of concentrations, repeating the experiments above and the inhibition rate was calculated respectively. The EC_50_ values were calculated by SPSS statistics v17.0 and the results are illustrated in Table 2

## 4. Conclusions

In conclusion, a series of novel 1, 2, 4-triazole fungicidal compounds with an oxime ether and phenoxypyridinyl moiety were designed and synthesized. The in vitro fungicidal activities against eight fungal pathogens were evaluated. Most of the compounds exhibited moderate to excellent fungicidal activities against tested phytopathogens. Especially, compound **5a4** displayed promising fungicidal activity with broad spectrum and **5b2** provided the highest inhibition rate towards *S. sclerotiorum*. In addition, other compounds towards certain fungus also exhibited high fungicidal activities, for example **5a17** against *B. cinerea*. and **5a18** against *P. infestans* and *R. solani*, Homology modeling and molecular docking disclosed the possible binding mode of the title compound in the SsCYP51 active site. Hydrophobic interactions in this class of compounds may play much more important role in enhancing the fungicidal activity. This work provided useful guidance for the design of new 1, 2, 4-triazole fungicides.

## Figures and Tables

**Table 1:** In vitro fungicidal activity against the phytopathogens ^a^.

Compds.	Mycelium Growth Inhibitory Rate (%) at 50 mg/L ^b^							
	FG	SS	BC	PI	PG	RS	FO	AA
**5a1**	44.8	50.2	22.9	69.0	72.7	62.7	49.1	29.2
**5a2**	59.0	89.0	44.8	61.7	46.6	71.3	51.8	29.6
**5a3**	55.7	85.4	32.6	64.2	48.6	74.5	52.9	24.0
**5a4**	51.5	92.3	75.6	75.2	42.7	77.9	58.5	41.9
**5a5**	27.3	88.0	81.0	65.0	52.8	70.7	49.8	52.5
**5a6**	30.1	24.0	20.7	36.6	32.2	47.8	61.1	25.6
**5a7**	44.8	90.7	72.5	60.6	43.9	64.9	45.6	38.3
**5a8**	38.8	44.0	62.8	60.5	37.7	71.3	56.5	52.1
**5a9**	66.7	83.1	49.6	59.7	67.8	68.2	63.8	55.1
**5a10**	45.1	62.2	30.9	74.5	62.6	68.2	64.2	31.4
**5a11**	39.9	88.7	70.5	58.7	47.0	75.9	47.9	37.6
**5a12**	37.8	73.7	66.7	42.9	24.1	45.3	40.9	35.2
**5a13**	55.2	27.9	12.8	76.4	27.6	51.1	59.6	26.2
**5a14**	48.1	87.8	47.0	74.6	61.4	79.7	63.1	32.6
**5a15**	42.1	88.7	32.1	72.0	53.3	75.9	59.6	25.9
**5a16**	37.6	79.8	0.0	57.2	56.7	71.8	58.0	23.2
**5a17**	40.3	83.7	78.3	52.5	57.6	78.6	73.1	26.6
**5a18**	37.6	84.3	43.5	74.6	19.8	82.7	48.4	21.4
**5b1**	45.1	65.1	47.2	48.4	57.2	42.4	40.6	30.4
**5b2**	49.0	90.0	47.2	36.1	43.9	36.9	43.7	18.7
**5b3**	45.2	76.8	39.9	39.5	43.1	36.6	38.3	15.1
**5b4**	0.0	43.8	16.1	0.0	0.0	19.1	7.9	1.5
**5b5**	26.6	45.2	30.2	20.6	7.3	22.1	18.0	7.2
**5b6**	18.7	27.0	35.3	11.5	0.0	27.2	16.0	0.0
**5b7**	30.3	73.4	33.6	27.4	35.4	34.4	26.4	17.2
**5b8**	28.1	70.4	14.3	21.8	12.8	26.4	18.8	16.2
**5b9**	39.1	82.3	31.7	46.2	33.3	35.6	32.8	26.3
**Difenoconazole**	93.7	99.3	90.2	86.0	99.5	89.3	94.2	84.3

^a^ Values are the mean of three replicates. ^b^ FG, Fusarium graminearum; SS, Sclerotinia sclerotiorum; BC, Botrytis cinerea; PI, Phytophthora infestans; PG, Pyricularia grisea; RS, Rhizoctonia solani; FO, Fusarium oxysporum; AA, Apsicum anthracnose.

**Table 2:** EC_50_ values of some compounds against four fungi.

Compds.	EC_50_ (mg/L)			
	SS	PI	RS	BC
**5a2**	4.40	29.87	10.43	58.90
**5a3**	10.07	18.64	9.80	71.56
**5a4**	1.59	0.46	0.27	11.39
**5a5**	4.71	26.78	34.25	19.68
**5a7**	4.30	19.09	11.95	46.25
**5a14**	17.47	5.92	19.33	37.04
**5a15**	2.64	17.93	15.43	67.78
**5a17**	3.65	41.46	65.41	7.48
**5a18**	15.09	3.02	5.25	14.50
**5b2**	0.12	>100	>100	89.05
**Difenoconazole**	0.02	0.26	0.09	8.86

SS, Sclerotinia sclerotiorum; PI, Phytophthora infestans; RS, Rhizoctonia solani; BC, Botrytis cinereal.

## Supplementary Materials

The following are available online. Figures are ^1^H NMR and ^13^C NMR of the synthesized compounds.
