# Supplementary material for: Design, Synthesis and Fungicidal Activity of New 1,2,4-Triazole Derivatives Containing Oxime Ether and Phenoxyl Pyridinyl Moiety
Source: Molecules. 2020 Dec 11;25(24):5852. doi: 10.3390/molecules25245852 (PMC7763646; doi:10.3390/molecules25245852)
Supplement: Supplementary file 1 [file molecules-25-05852-s001.pdf]

Supplementary materials

Design, Synthesis and Fungicidal Activity of Novel 1, 2, 4-Triazole Derivatives Containing Oxime Ether and Phenoxylpyridinyl Moiety

Hui Bai, Xuelian Liu, Pengfei Chenzhang, Jiaqi Li, Yumei Xiao, Bin Fu,* Zhaohai Qin*

Table of Contents

1. Crystal structure determination……………………………………………….S2

2. Fungicidal activity assay……………………………………………………….S3

3. Homology modeling and molecular docking…………………..……………….S3

4. References……………………………………………………..………………..S5

5. The spectra of ^{1}HNMR and ^{13}CNMR.........................…...……………...……S6

* Corresponding authors.

E-*mail addresses*: fubinchem@cau.edu.cn (B. Fu), qinzhaohai@263.net (Z. Qin).

1. Crystal structure determination for compound **5a18**

Compound **5a18** was recrystallized by slow evaporation from a mixed solvent of methanol/ethyl acetate (1:4 v/v) to afford crystals suitable for X-ray diffraction analysis. Colorless pieces of **5a18** were mounted on a quartz fiber. Cell dimensions and intensities were measured using a Bruker D8 Venture X-ray CMOS diffractometer with graphite monochromated MoKa radiation. The structure was resolved by direct methods with SHELXS-97. Hydrogen atoms were observed and refined at a fixed value of their isotropic displacement parameter. Crystallographic data for the structure of compound **5a18** have been deposited in the Cambridge Crystallographic Data Centre (deposition number CCDC-2015868).

Table S1. Crystal data and structure refinement for compound **5a18**.

Empirical formula	$C_{16}H_{14}N_6O_4$
Formula weight	354.33
Temperature	298 K
Wavelength	0.71073 Å
Crystal system, space group	Monoclinic, *P2*(1)/n
Unit cell dimensions	*a* = 11.0892(16) Å α = 90°.
	b = 4.1671(6) Å β = 98.993(5)°.
	c = 18.184(3) Å γ = 90°.
Volume	830.0(2) $Å^3$
Z, Calculated density	2, 1.418 Mg/m^3
Absorption coefficient	0.106 mm^{-1}
F(000)	368.0
h,k,lmax	14,5,23
Data completeness	1.73/0.98
Theta(max)	27.436
R(reflections)	0.0589(2488)
wR2(reflections)	0.1784(3744)
S	1.032
Npar	237

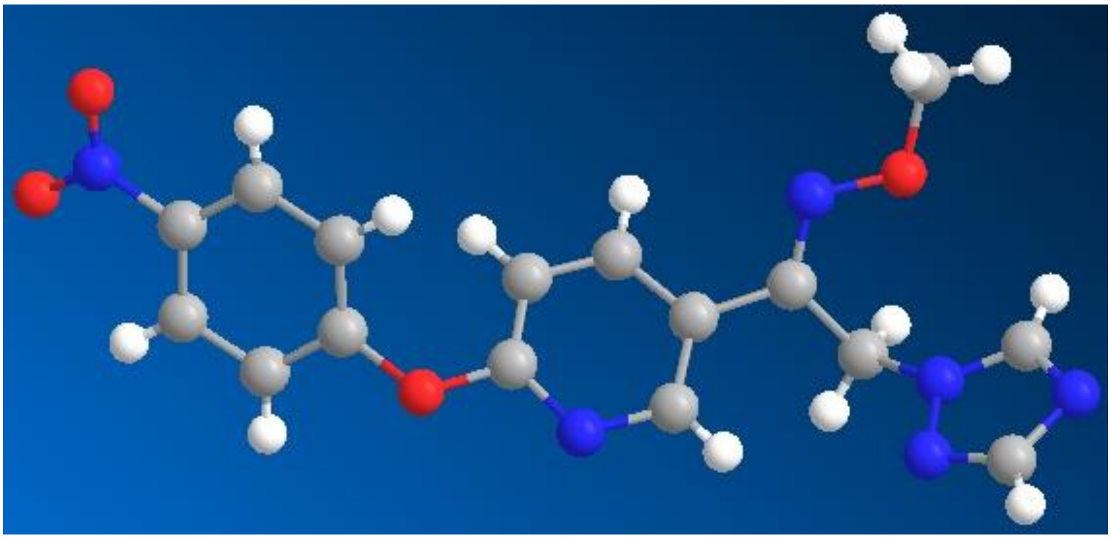

The structure of **5a18**

2. Fungicidal Activity assay (Growth Inhibition Test).

The in vitro fungicidal activities against plant pathogens were tested according to the reported method[2]. The medium was amended with aliquots of each tested compound solution to provide a concentration of 50 mg/L. The tested compounds were dissolved in 0.3 mL of dimethyl sulfoxide (DMSO) and added aseptically to molten agar after autoclaving, when the agar had cooled to 45−50 °C. The concentration of solvent never exceeded 0.1 mg/L. The mixed medium without sample was used as the blank control. The inocula, 5 mm in diameter, were removed from the margins of actively growing colonies of mycelium, placed in the centers of the above plates. Three replicates were done for each concentration, and the control plates were sealed with parafilm and incubated at 26 °C in darkness. The diameter of the mycelium was measured after several days. The inhibition percent was used to describe the control efficiency of the compounds. Inhibition percent (%) = (hyphal diameter in the control − hyphal diameter in the treatment)/hyphal diameter in the control. The results are summarized in Table S1.

A 20 mg/mL stock solution was diluted with PDA to obtain a series of concentrations, repeating the experiments above, and the inhibition rate was calculated respectively. The EC_{50} values were calculated by spss statistics v17.0 , and the results are illustrated in Table 1

3. Homology modeling and molecular docking

Homology modeling was carried out with the online program SWISS MODEL (http://swissmodel.expasy.org/). First, the primary amino acid sequence of CYP51 (Sclerotinia sclerotiorum organism) was uploaded to the program, then the SWISS-MODEL template library (SMTL version 2020-04-08, PDB release 2020-04-03) was searched with BLAST[3] and HHBlits[4] for evolutionary related structures matching the uploaded sequence (target sequence) when the

Search For Templates was performed. Next, according to the reported result, the template was chosen for homology modelling of CYP51 crystal structure. Model was built based on the target-template alignment using ProMod3. Coordinates which are conserved between the target and the template are copied from the template to the model. Insertions and deletions are remodelled using a fragment library. Side chains are then rebuilt. Finally, the geometry of the resulting model is regularized by using a force field. In case loop modelling with ProMod3 fails, an alternative model is built with PROMOD-II . The global and perresidue model quality has been assessed using the QMEAN scoring function[5]. The built model was selected for the following molecular docking simulation. The templates for CYP51 was 6CR2[6].

Molecular docking simulations of compounds (**5a4**, **5b2** and **difenoconazole**) against CYP51 were performed with Glide program (Schrö dinger, LLC: New York, NY, 2015)[7]. The protein structures were prepared using the Protein Preparation Wizard panel inserted in the Maestro with the default settings. Residues within 20 Å around the ligand were defined as binding sites in which the docking grids were generated by the Receptor Grid Generation panel. The default settings were adopted for the cutoff, neutralization, etc.. Finally, the prepared compounds were docked to the aforementioned docking girds with Standard precision (SP) mode. The top ranking pose of each compound was respectively selected for binding mode analysis.

```
Target     MGILETIAGPLAQEISQRSTFAVVAAGVAAFVVLSVILNVLNQVLFANPNEPPVVFHWFPIIGSTVTYGMDPYKFFFECR
6cr2.1.A   ---------------------------------------------------PPVVFHWFPFIGSTISYGIDPYKFFFDCR

Target     AKYGDIFTFVLLGKKNTVYLGRNGNDFILNGKLKDLNAEEIYTVLTTPVFGKDVVYDCPNAKLMEQKKFMKIGLSTEAFR
6cr2.1.A   AKYGDIFTFILLGKKTTVYLGTKGNDFILNGKLRDVCAEEVYSPLTTPVFGRHVVYDCPNAKLMEQKKFVKYGLTSDALR

Target     SYVPIIQMEVENFMKRSSVFKGQKGTADIGPAMAEITIYTASHTLQGKEVRDRFDTTFASLYHDLDMGFSPINFMLHWAP
6cr2.1.A   SYVPLITDEVESFVKNSPAFQGHKGVFDVCKTIAEITIYTASRSLQGKEVRSKFDSTFAELYHNLDMGFAPINFMLPWAP

Target     LPHNRARDHAQRTVAATYMDIIKKRRAQATEADFKSDIMWQLMRSSYKDGTPVPDREIAHMMIALLMAGQHSSSSSISWI
6cr2.1.A   LPHNRKRDAAQRKLTETYMEIIKARRQAGSKKD-SEDMVWNLMSCVYKNGTPVPDEEIAHMMIALLMAGQHSSSSTASWI

Target     LLRLASRPDIMEELYQEQIQVLGADLPALKYEDLAKLPLHQNILKETLRIHTPIHSIMRKVTTPMPISGTKYVIPTSHTL
6cr2.1.A   VLRLATRPDIMEELYQEQIRVLGSDLPPLTYDNLQKLDLHAKVIKETLRLHAPIHSIIRAVKNPMAVDGTSYVIPTSHNV

Target     MASPGCTSRDADYFPEPLEWDPHRWDIGSGRVIGNDQDEEFQDYGYGMISKGASSPYLPFGAGRHRCIGEQFANVQLITI
6cr2.1.A   LSSPGVTARSEEHFPNPLEWNPHRWD---ENIAASAEDDEKVDYGYGLVSKGTNSPYLPFGAGRHRCIGEQFAYLQLGTI

Target     MATVVRMFKFKNVDGSKDVIGTDYTSLFTRPLAPAVIAWERR
6cr2.1.A   TAVLVRLFRFRNLPGVDGIPDTDYSSLFSKPLGRSFVEFEKR
```

Figure S1. The alignment of target-template sequence

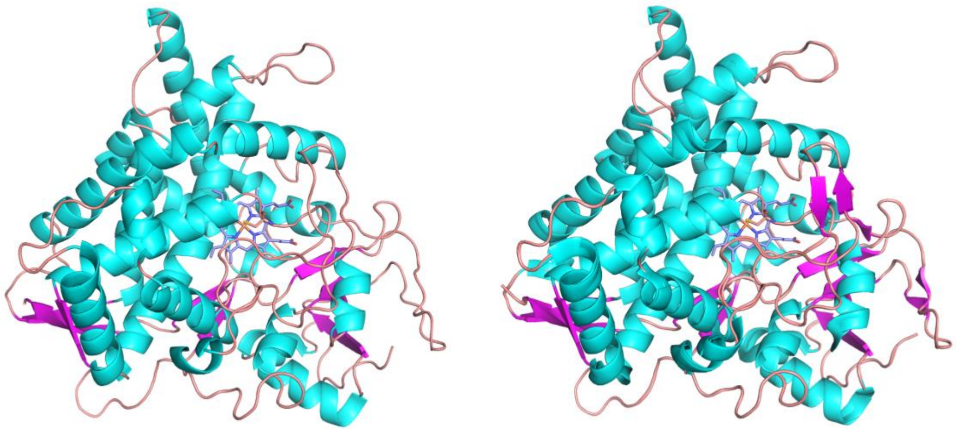

A B

Figure S2. A: The modeled structure of CYP51 of *S. sclerotiorum in cartoon diagram.*

B: The modeled structure of CYP51 of *S.sclerotiorum* in cartoon diagram was superposed with the template structure.

The α-helix, β sheet and loop were colored in cyan, magenta and salmon, respectively. The heme was shown as sticks representation.

4. REFERENCES

1. Qin, Z.; Yang, D. High-activity imino phenylacetate compounds and preparation method and application thereof. CN106946770A, 2017.
2. Cao, X.; Fei, L.; Ming, H.; Lu, W.; Sheng, H. L., Chiral γ-Aryl-1 H -1,2,4-triazole Derivatives as Highly Potential Antifungal Agents: Design, Synthesis, Structure, and in Vitro Fungicidal Activities. *J. Agri. Food Chem.* 2008, *56* (23), 11367-11375.
3. Camacho, C.; Coulouris, G.; Avagyan, V.; Ma, N.; Papadopoulos, J.; Bealer, K.; Madden, T. L., BLAST+: architecture and applications. *BMC Bioinformatics* 2009, *10* (1), 421-430.
4. Remmert, M.; Biegert, A.; Hauser, A.; Söding, J., HHblits: Lightning-fast iterative protein sequence searching by HMM-HMM alignment. *Nature Methods* 2012, *9* (2), 173-175.
5. Studer, G., Rempfer, C., Waterhouse, A.M., Gumienny, G., Haas, J., Schwede, T. QMEANDisCo - distance constraints applied on model quality estimation. Bioinformatics 2020, 36, 1765-1771.
6. Friggeri, L., Hargrove, T.Y., Wawrzak, Z., Blobaum, A.L., Rachakonda, G., Lindsley, C.W., Villalta, F., Nes, W.D., Botta, M., Guengerich, F.P., Lepesheva, G.I. Sterol 14 alpha-Demethylase Structure-Based Design of VNI ((R)- N-(1-(2,4-Dichlorophenyl)-2-(1 H-imidazol-1-yl)ethyl)-4-(5-phenyl-1,3,4-oxadiazol-2-yl)benzamide)) Derivatives To Target Fungal Infections: Synthesis, Biological Evaluation, and Crystallographic Analysis. J Med Chem 2018, 61: 5679-5691.
7. Halgren, T. A.; Murphy, R. B.; Friesner, R. A.; Beard, H. S.; Frye, L. L.; Pollard, W. T.; Banks, J. L., Glide: A New Approach for Rapid, Accurate Docking and Scoring. 2. Enrichment Factors in Database Screening. *J. Med. Chem.* 2004, *47* (7), 1750-1759.

5. The spectra of ^{1}HNMR and ^{13}CNMR

1-(6-chloropyridin-3-yl)ethan-1-one **(1)**

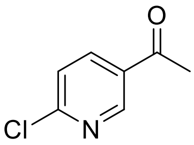

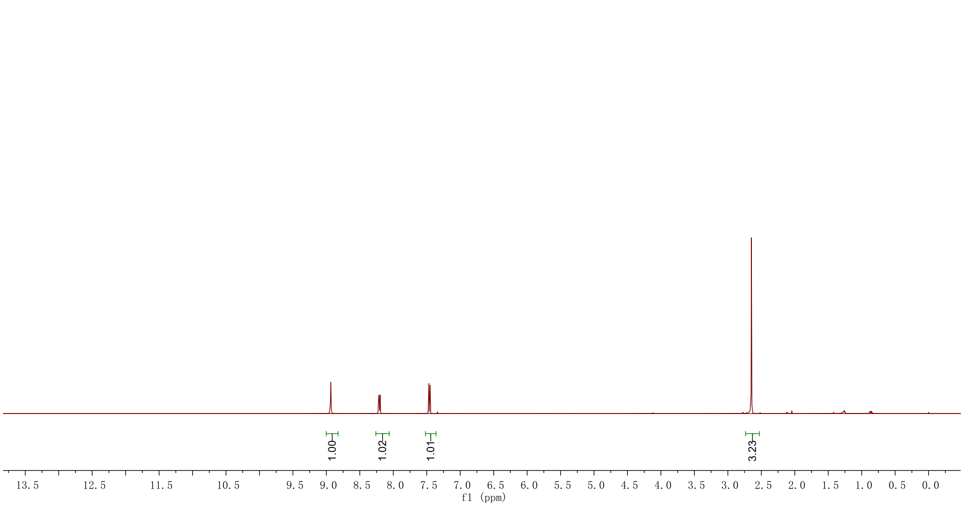

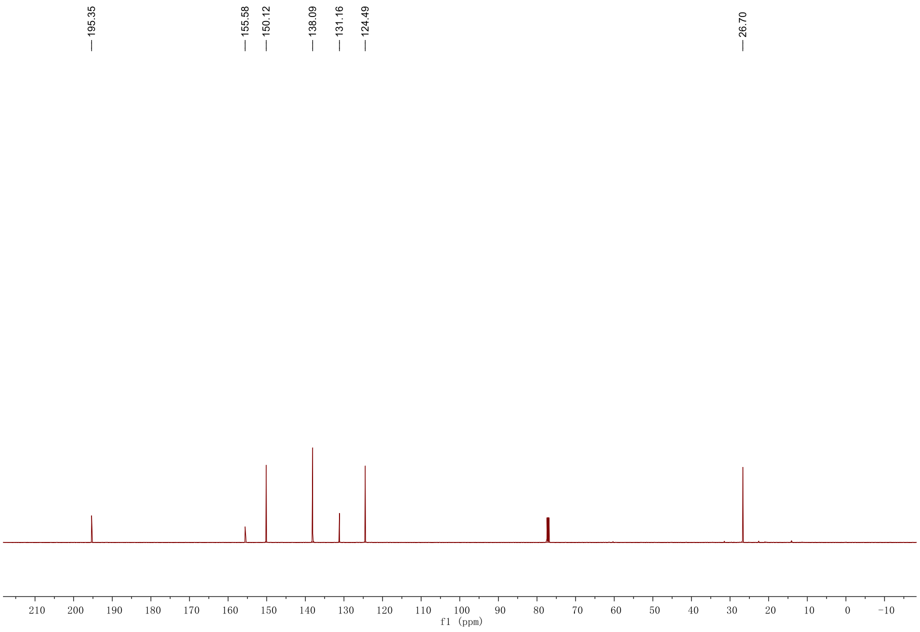

2-bromo-1-(6-chloropyridin-3-yl)ethan-1-one**(2)**

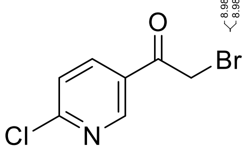

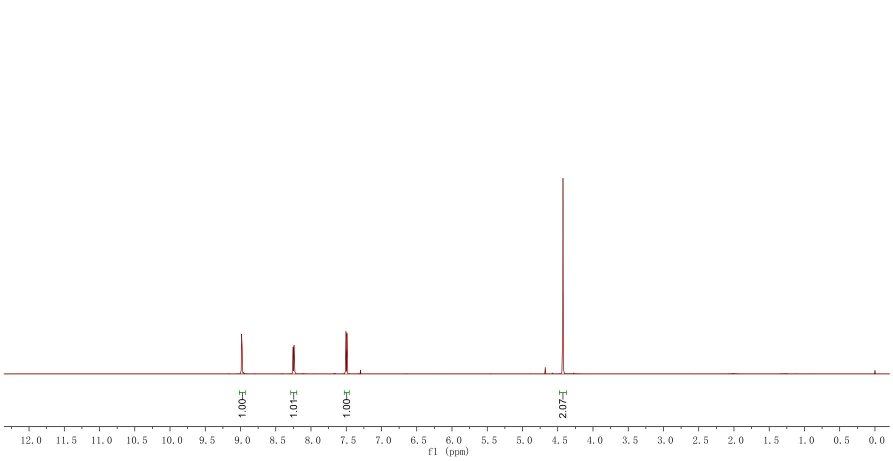

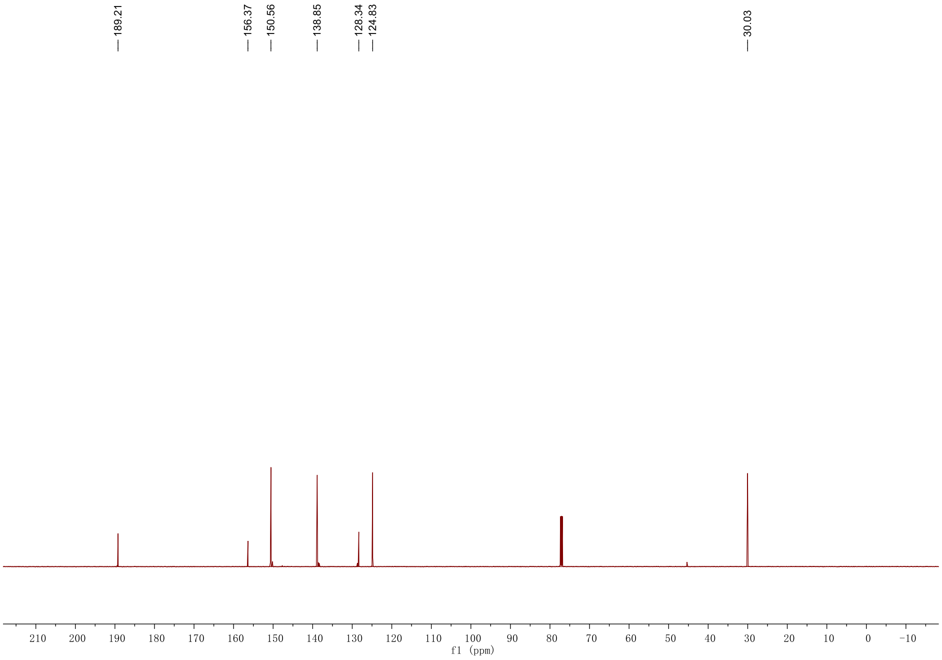

1-(6-chloropyridin-3-yl)-2-(1H-1,2,4-triazol-1-yl)ethan-1-one（3）

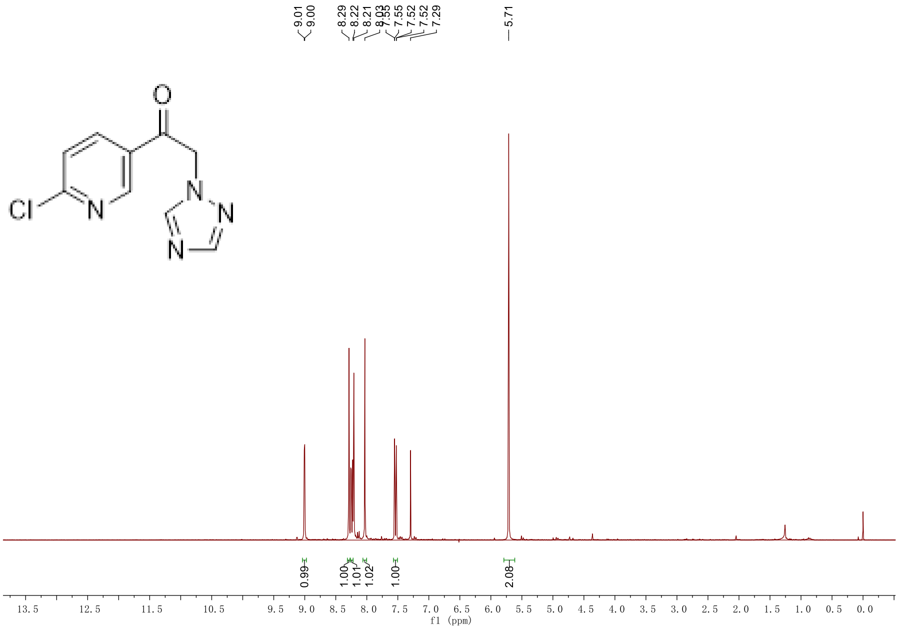

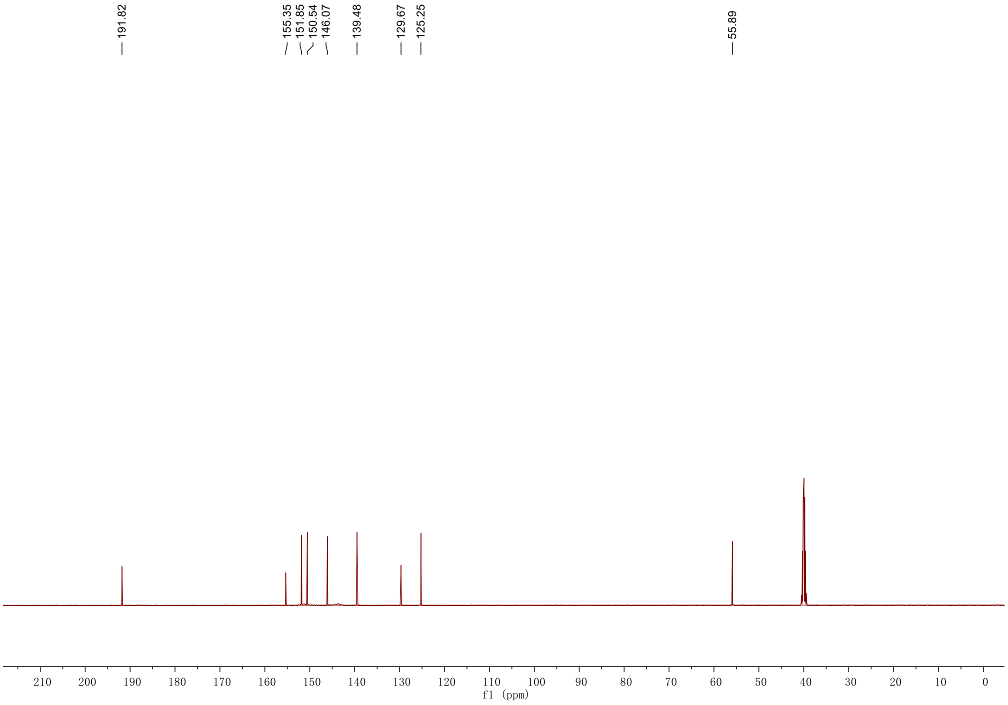

(Z)-1-(6-chloropyridin-3-yl)-2-(1H-1,2,4-triazol-1-yl)ethan-1-one O-methyl oxime **(4a)**

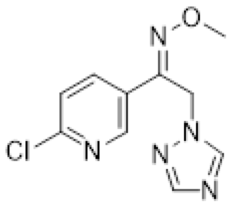

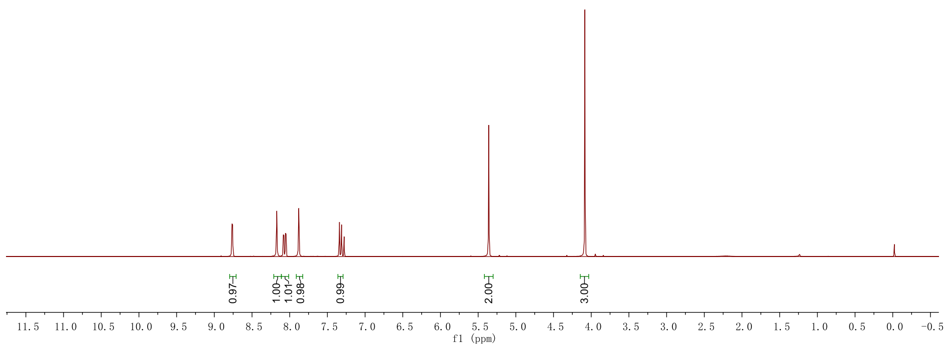

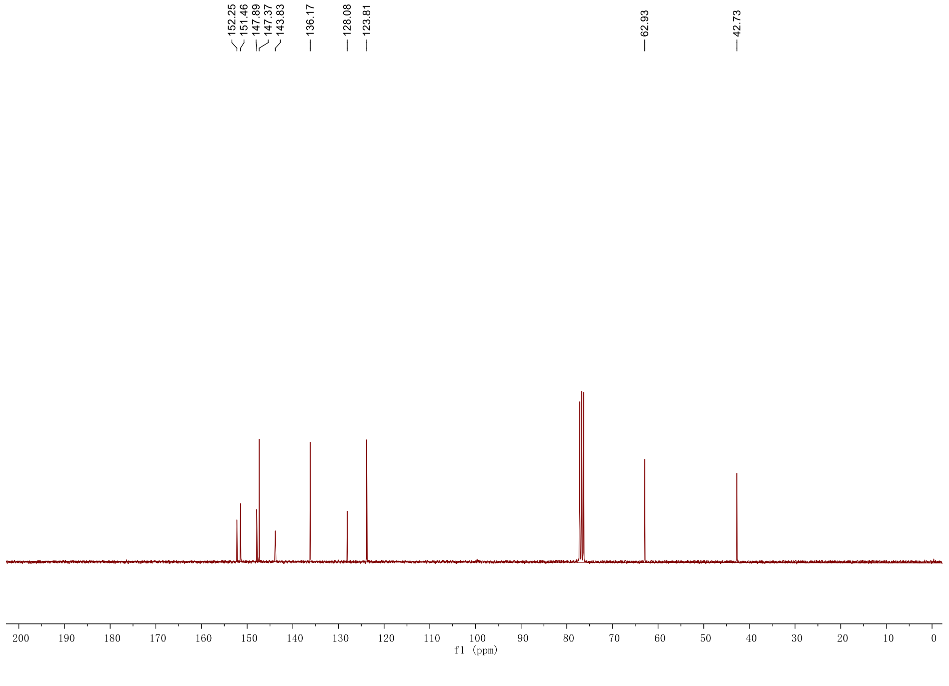

(Z)-1-(6-chloropyridin-3-yl)-2-(1H-1,2,4-triazol-1-yl)ethan-1-one O-benzyl oxime（**4b**）

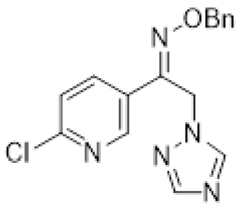

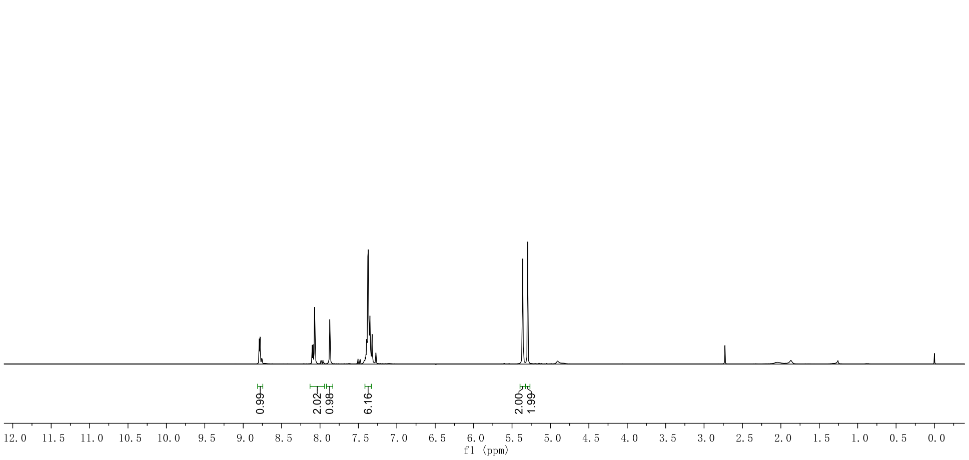

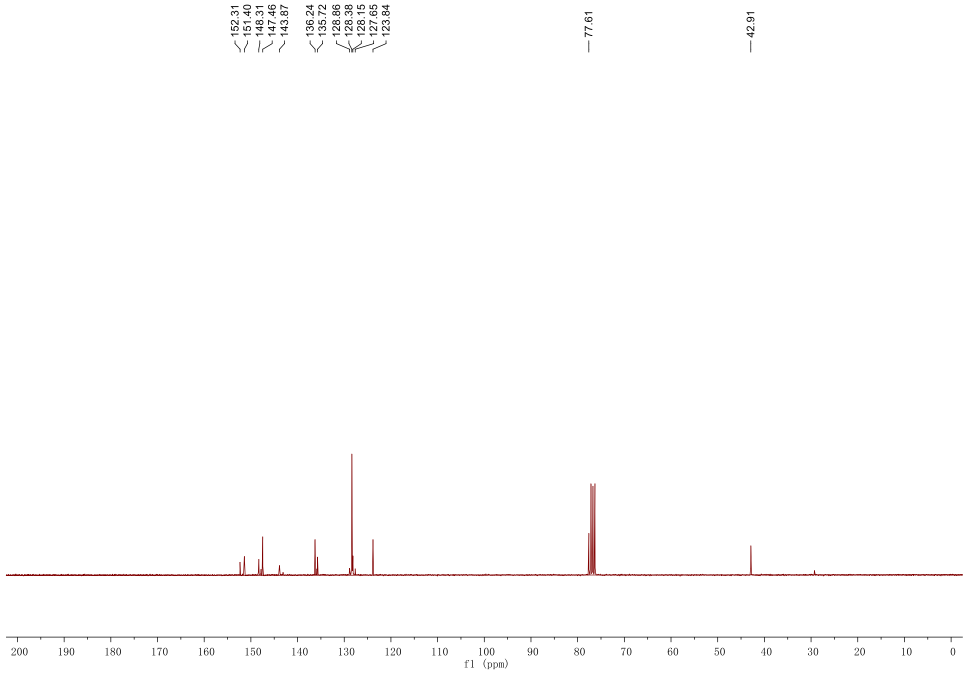

5a1

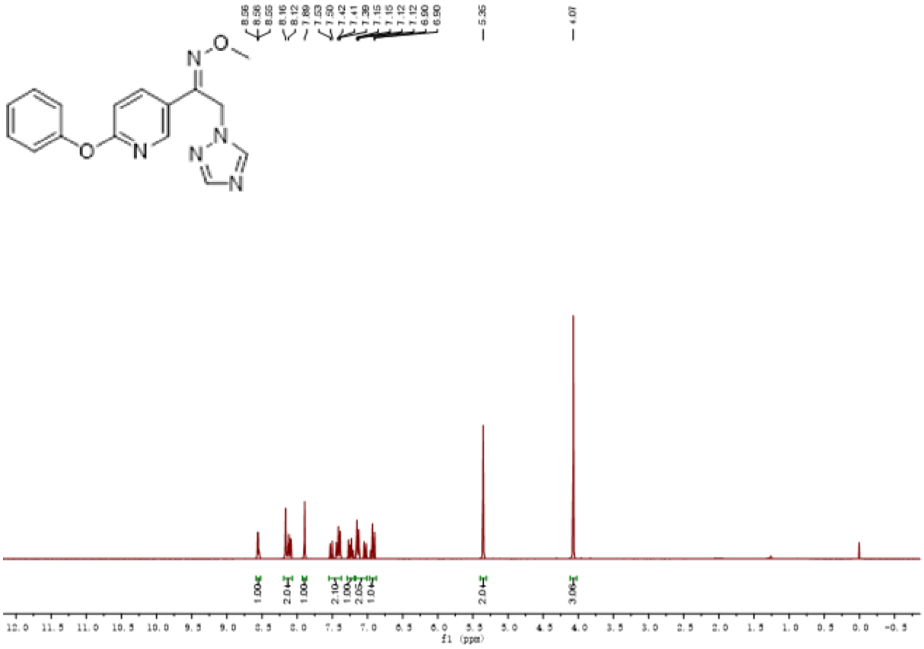
8.56
8.56
8.55
8.16
8.12
7.89
7.53
7.50
7.42
7.41
7.39
7.15
7.15
7.12
6.90
6.90
5.36
4.07
f1 (ppm)

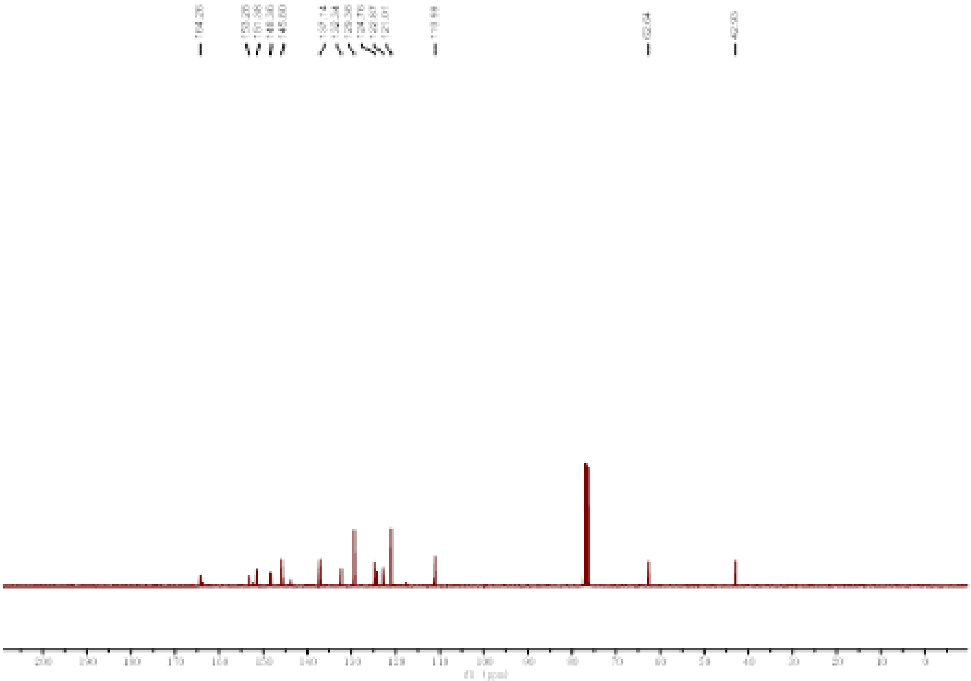

5a2

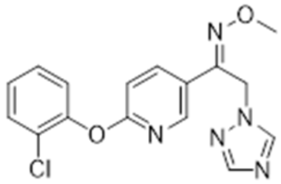
N
O
O
N
N
N
N
Cl

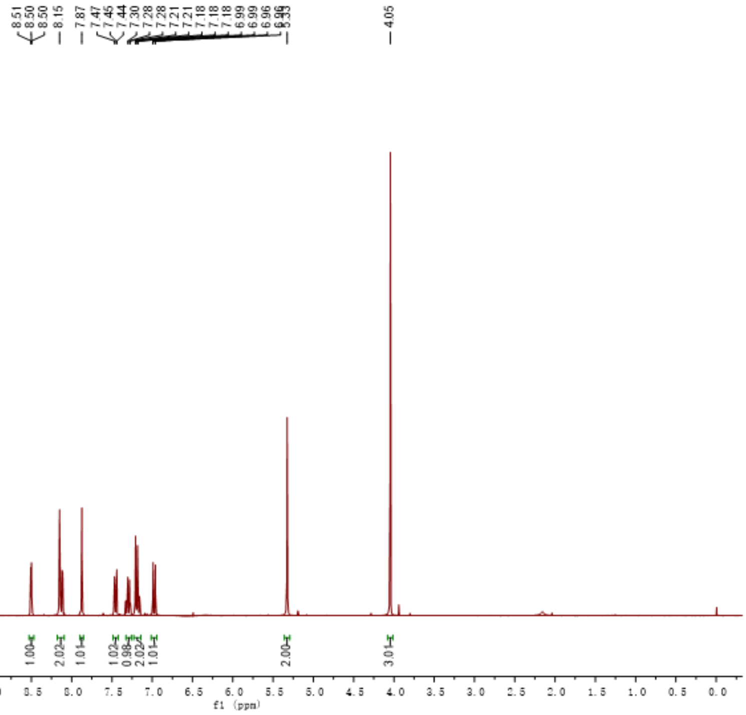
8.51
8.50
8.50
8.15
7.87
7.47
7.45
7.44
7.30
7.28
7.28
7.21
7.21
7.18
7.18
7.18
6.99
6.99
6.96
4.05
1.00
2.02
1.01
1.02
0.98
2.02
1.01
2.00
3.01
12.0 11.5 11.0 10.5 10.0 9.5 9.0 8.5 8.0 7.5 7.0 6.5 6.0 5.5 5.0 4.5 4.0 3.5 3.0 2.5 2.0 1.5 1.0 0.5 0.0
f1 (ppm)

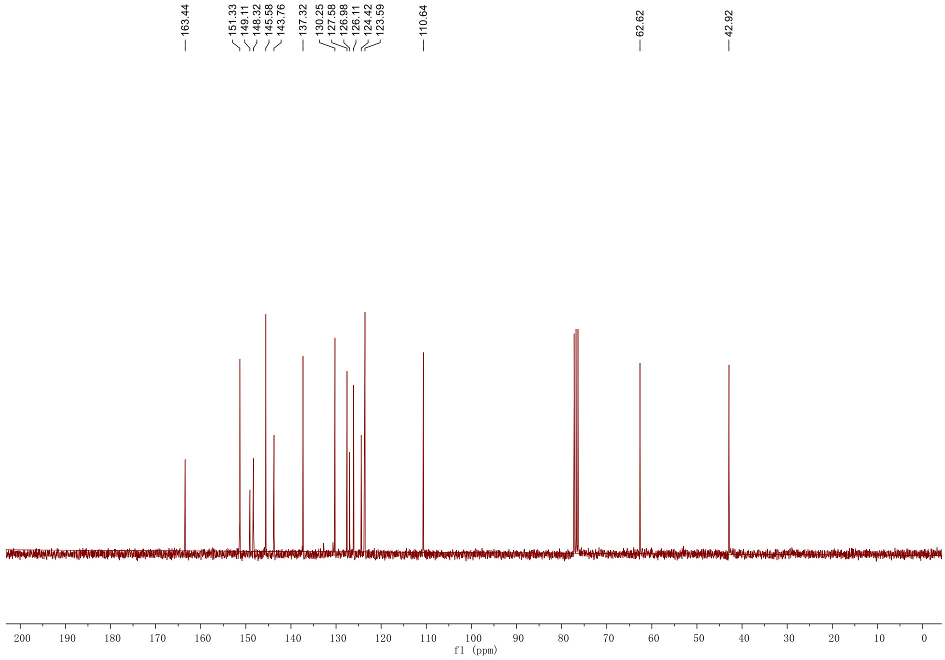
163.44
151.33
149.11
148.32
145.58
143.76
137.32
130.25
127.58
126.98
126.11
124.42
123.59
110.64
62.62
42.92
200 190 180 170 160 150 140 130 120 110 100 90 80 70 60 50 40 30 20 10 0
f1 (ppm)

5a3

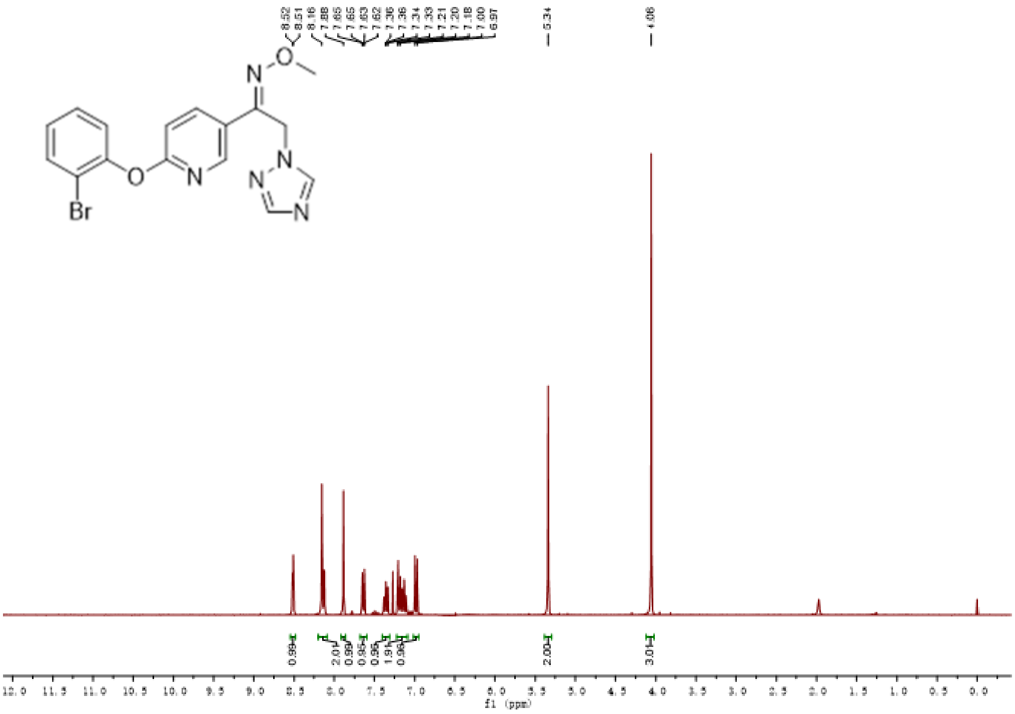
8.52
8.51
8.16
7.88
7.65
7.65
7.63
7.62
7.36
7.36
7.34
7.33
7.21
7.20
7.18
7.00
6.97
5.34
4.06
Br
O
N
N
O
N
N
N
0.99
2.01
0.99
0.93
0.95
1.91
0.96
2.00
3.01
f1 (ppm)

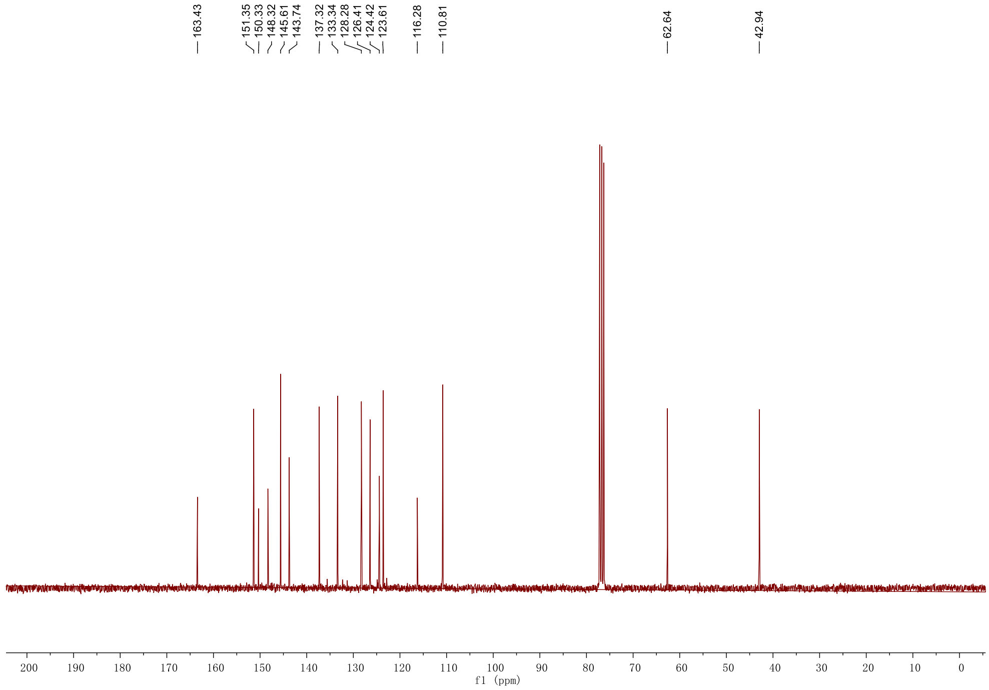
163.43
151.35
150.33
148.32
145.61
143.74
137.32
133.34
128.28
126.41
124.42
123.61
116.28
110.81
62.64
42.94
f1 (ppm)

5a4

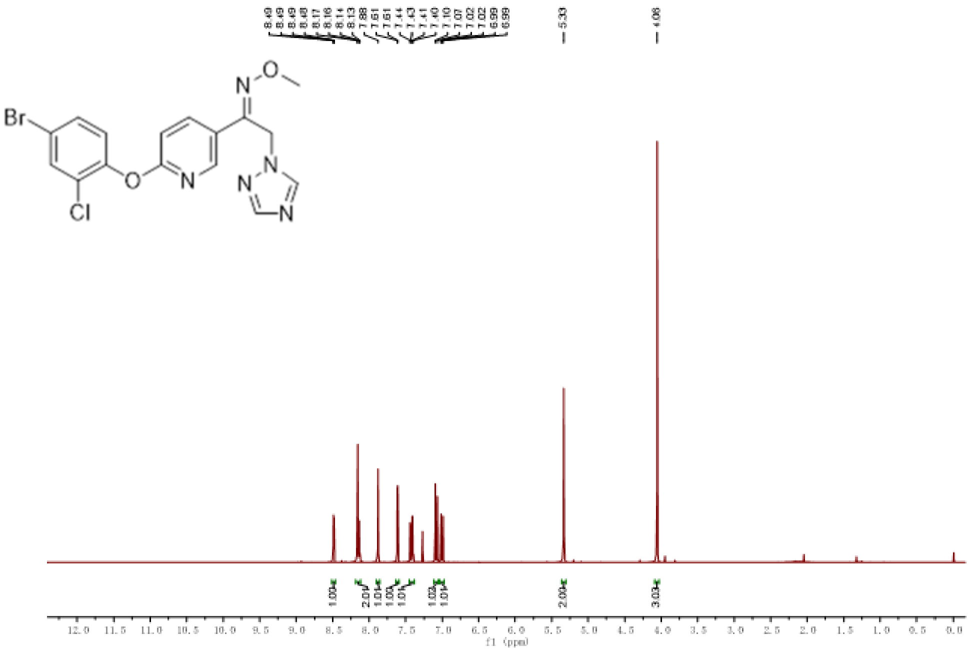
Br
Cl
O
N
N
O
N
N
N
8.49
8.49
8.49
8.48
8.17
8.16
8.14
8.13
7.88
7.61
7.61
7.44
7.43
7.41
7.40
7.10
7.07
7.02
7.02
6.99
6.99
5.33
4.08
1.00
2.01
1.01
1.00
1.01
1.02
1.01
2.00
3.03
f1 (ppm)

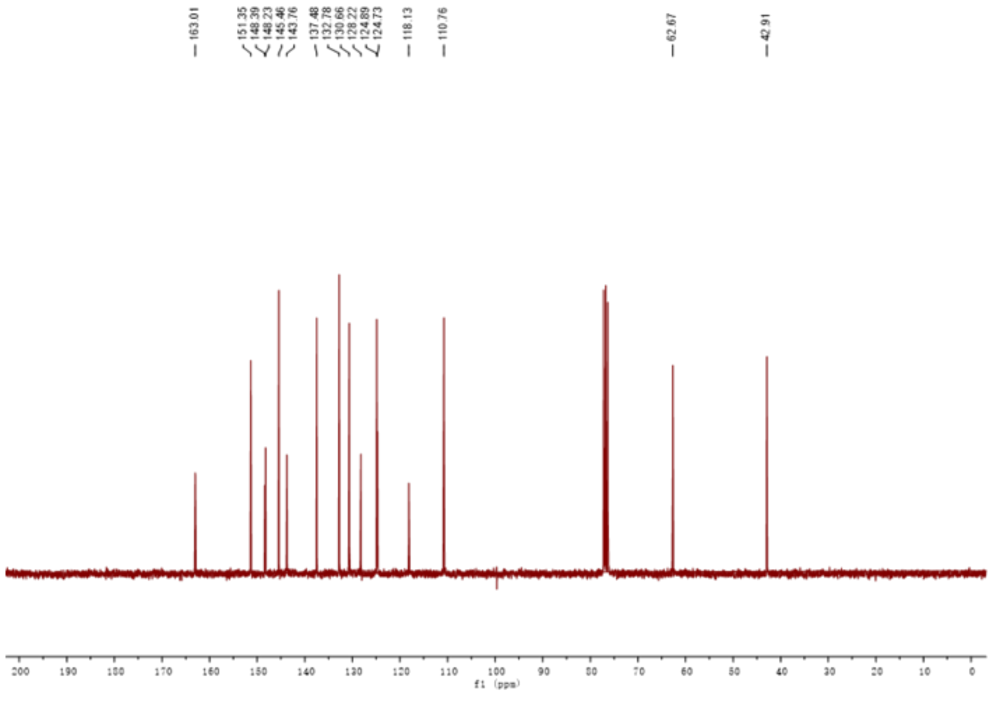
163.01
151.35
148.39
148.23
145.46
143.76
137.48
132.78
130.66
128.22
124.89
124.73
118.13
110.76
62.67
42.91
f1 (ppm)

5a5

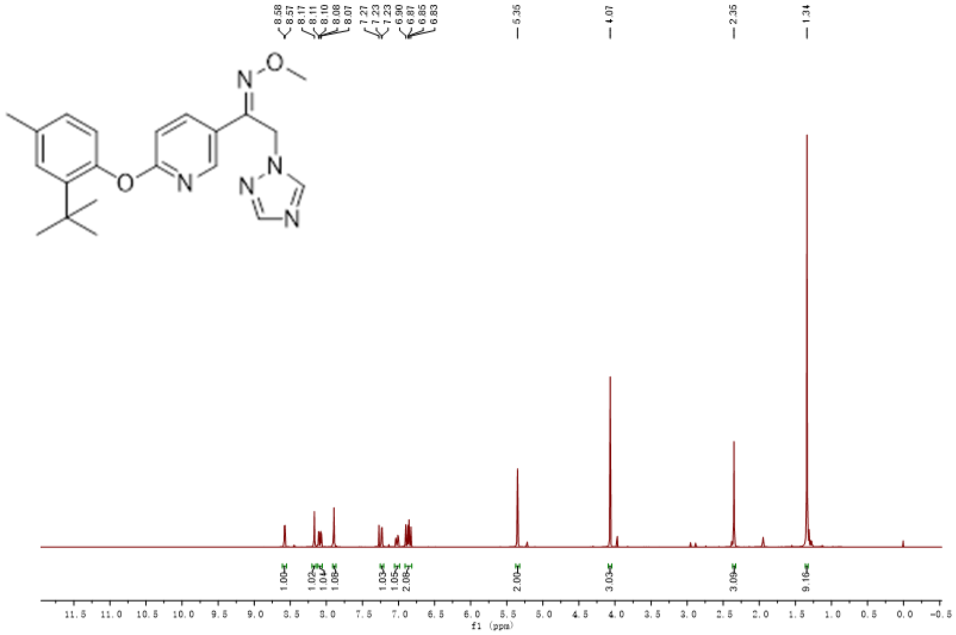
8.58
8.57
8.17
8.11
8.10
8.08
8.07
7.27
7.23
7.23
6.90
6.87
6.85
6.83
5.36
4.07
2.35
1.34
N
O
O
N
N
N
N
1.00
1.02
1.01
1.08
1.03
1.05
2.08
2.00
3.03
3.09
9.16
11.5
11.0
10.5
10.0
9.5
9.0
8.5
8.0
7.5
7.0
6.5
6.0
5.5
5.0
4.5
4.0
3.5
3.0
2.5
2.0
1.5
1.0
0.5
0.0
-0.5
f1 (ppm)

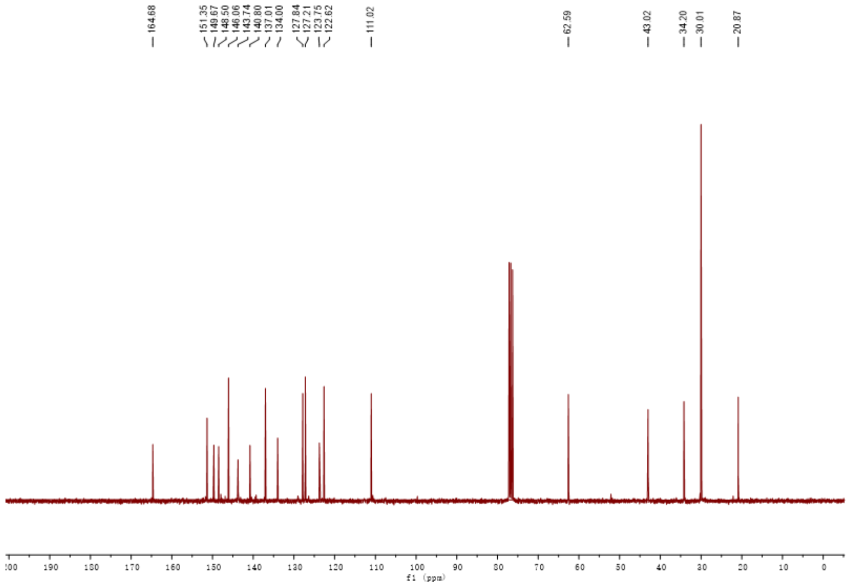
164.68
151.35
149.67
148.50
146.06
143.74
140.80
137.01
134.00
127.84
127.21
123.75
122.62
111.02
62.59
43.02
34.20
30.01
20.87
190
180
170
160
150
140
130
120
110
100
90
80
70
60
50
40
30
20
10
0
f1 (ppm)

5a6

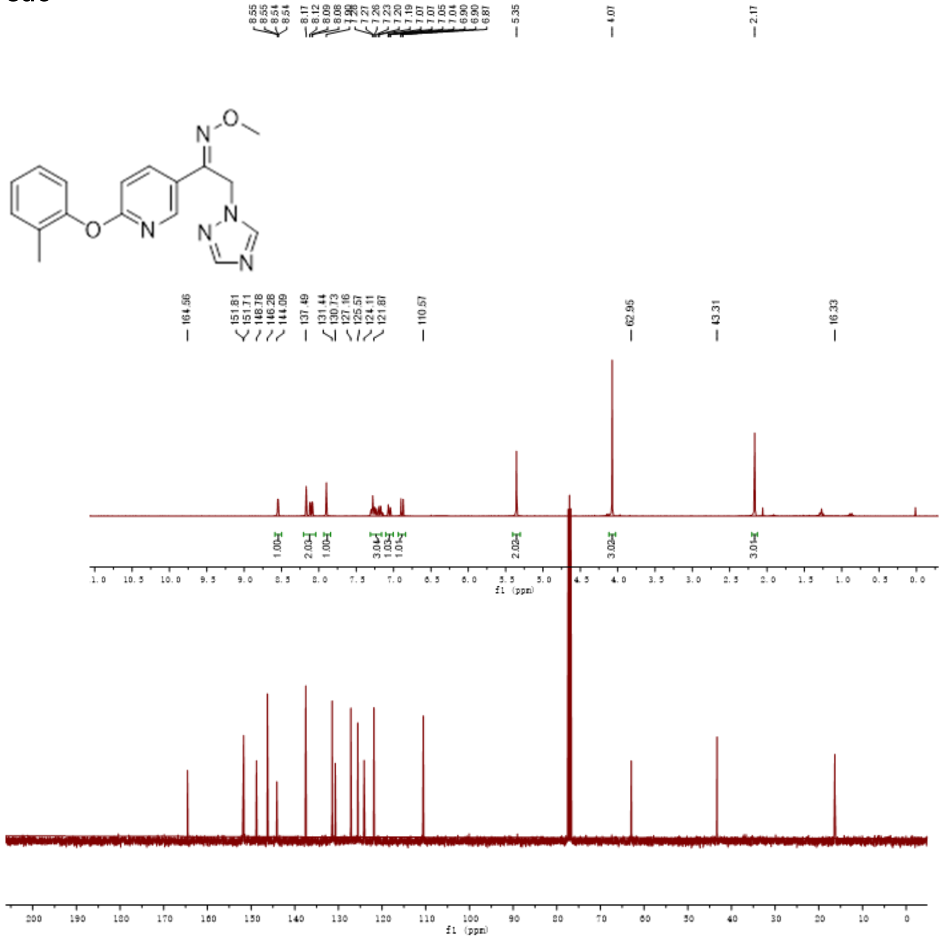

5.35
4.07
2.17
164.56
151.81
151.71
148.78
146.28
144.09
137.49
131.44
130.73
127.16
125.57
124.11
121.87
110.57
62.95
43.31
16.33
f1 (ppm)
f1 (ppm)

5a7

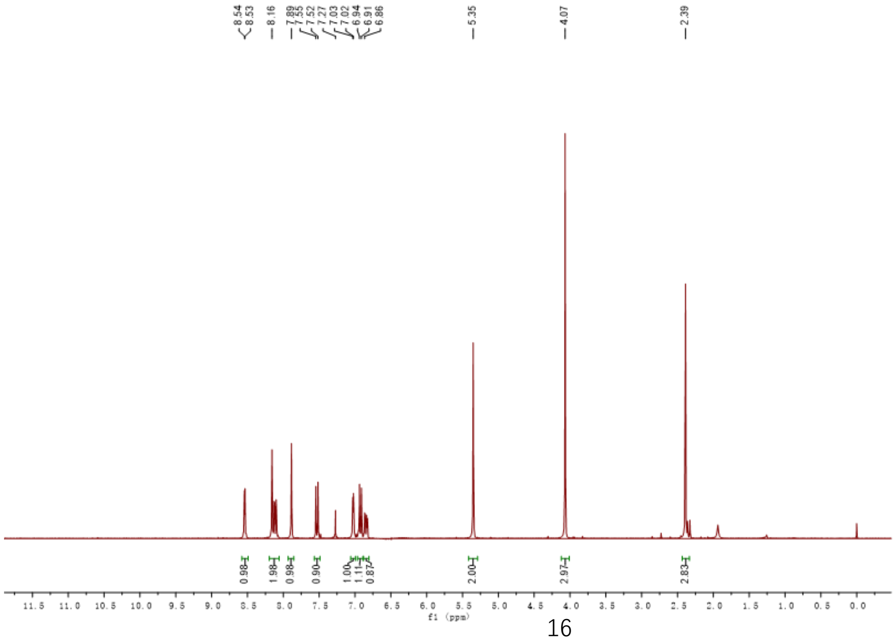

8.54
8.53
8.16
5.35
4.07
2.39
f1 (ppm)

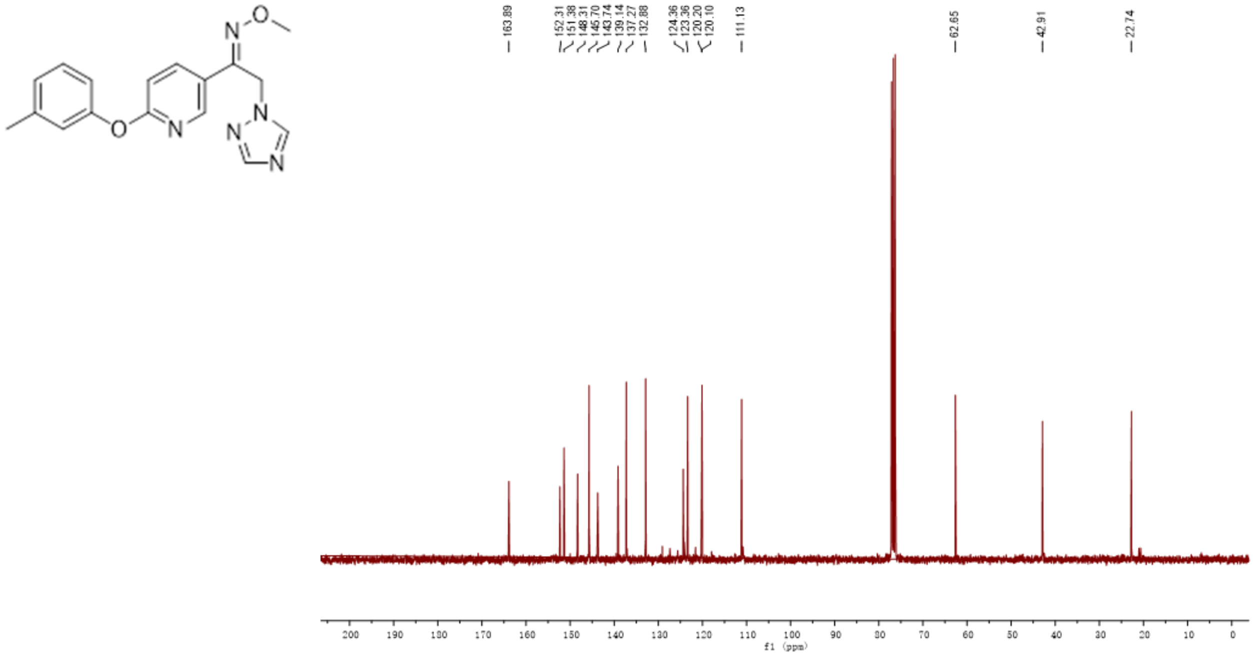
MeO
N
O
N
N
N
N
163.89
152.31
151.38
148.31
145.70
143.74
139.14
137.27
132.88
124.36
123.36
120.20
120.10
111.13
62.65
42.91
22.74
f1 (ppm)

5a8

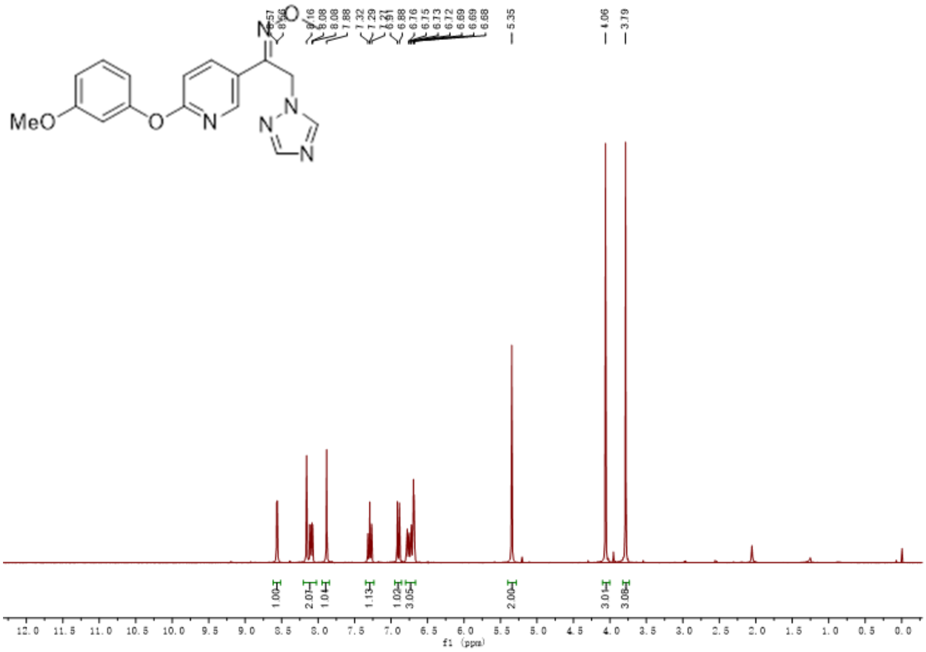
MeO
O
N
N
N
N
N
8.57
8.56
8.16
8.08
8.08
7.88
7.32
7.29
7.21
6.91
6.88
6.76
6.75
6.73
6.72
6.69
6.69
6.68
5.35
4.06
3.79
1.00
2.07
1.04
1.13
1.02
3.05
2.00
3.01
3.06
f1 (ppm)

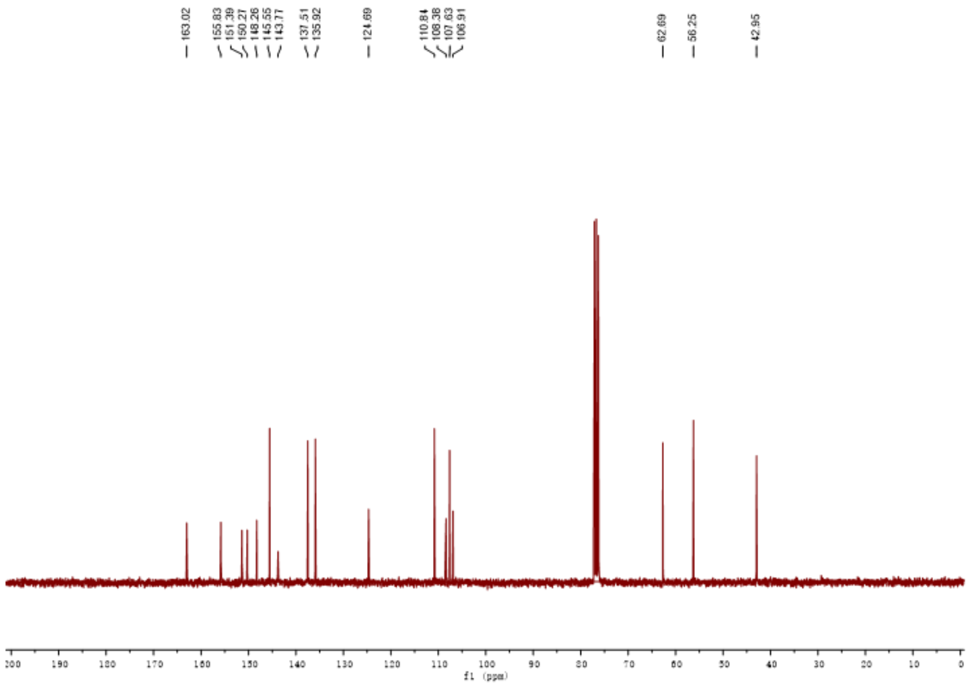

5a9

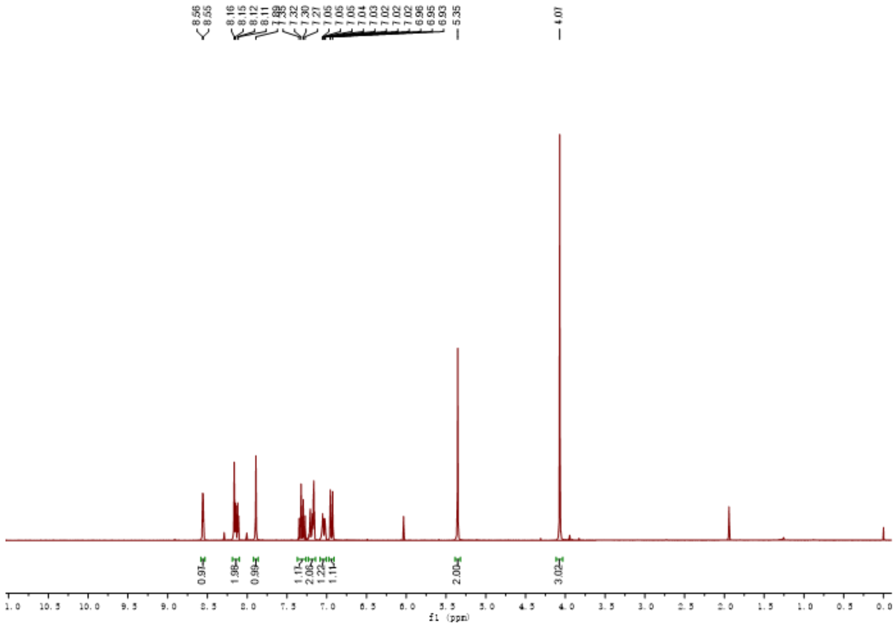

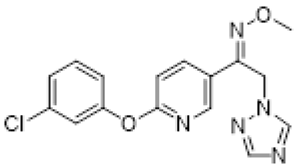
N
O
Cl
O
N
N
N
N

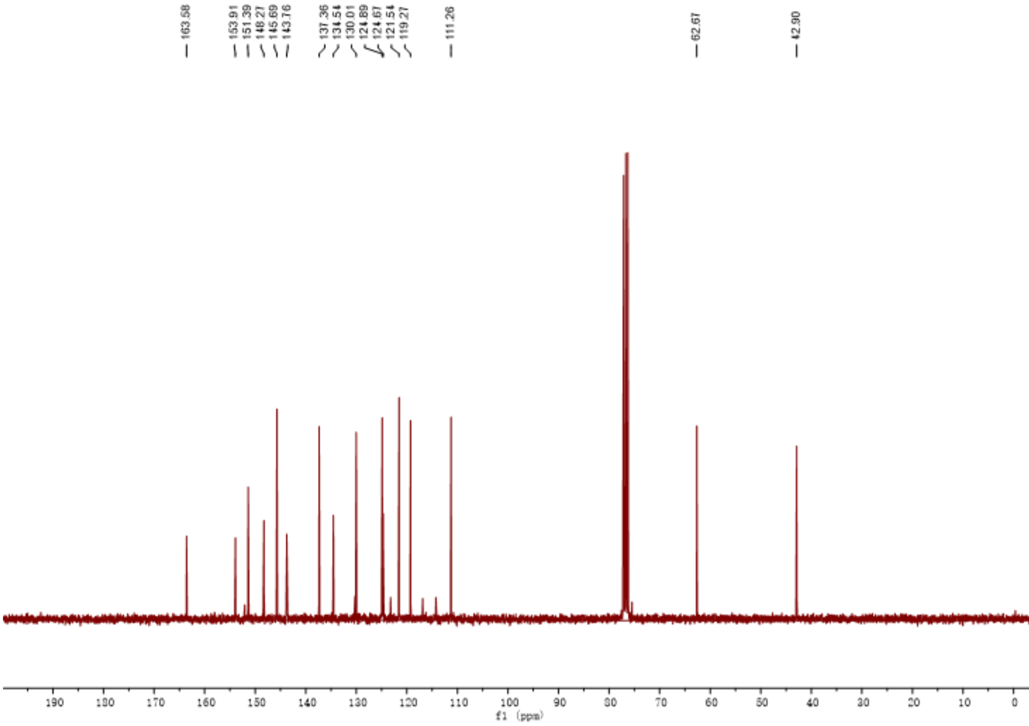
163.58
153.91
151.39
148.27
145.69
143.76
137.36
134.54
130.01
124.89
124.67
121.54
119.27
111.26
62.67
42.90
f1 (ppm)

5a10

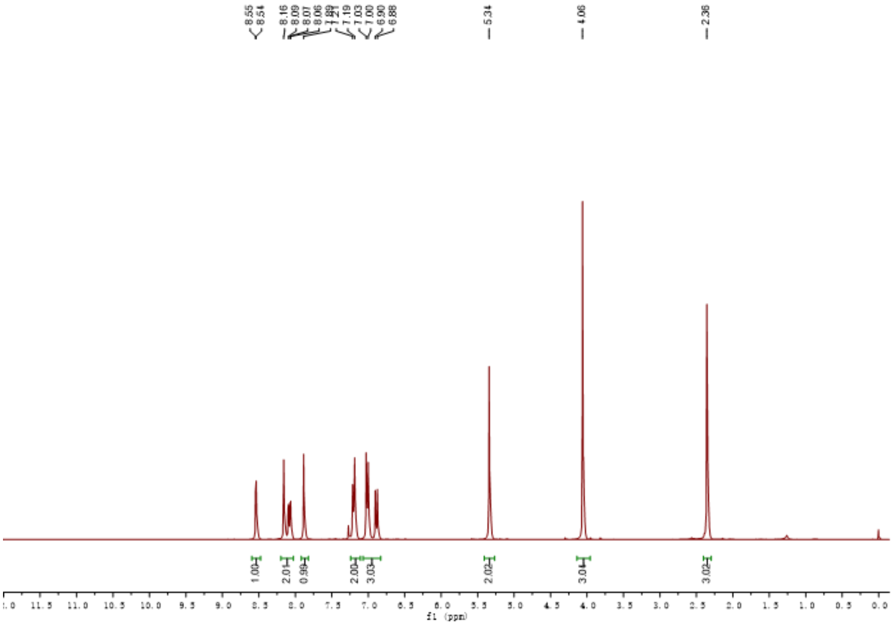
8.55
8.54
8.16
8.09
8.07
8.06
7.19
7.03
7.00
6.90
6.88
5.34
4.06
2.36
1.00
2.01
0.99
2.00
3.03
2.02
3.04
3.02
f1 (ppm)

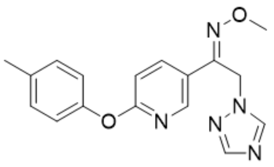

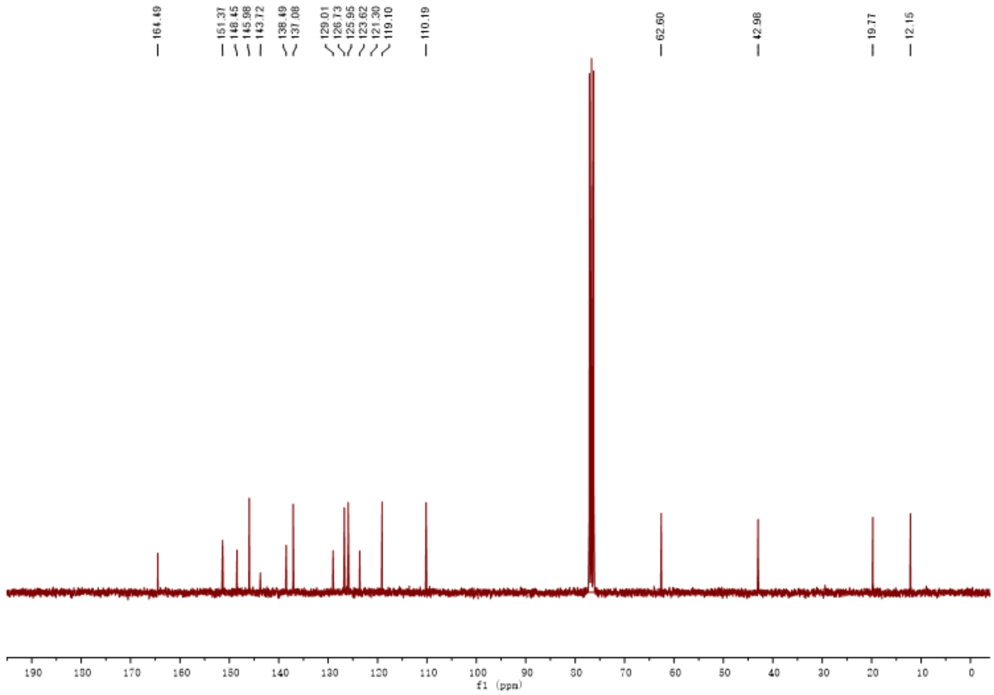
164.49
151.37
148.45
145.98
143.72
138.49
137.08
129.01
126.73
125.95
123.62
121.30
119.10
110.19
62.60
42.98
19.77
12.15
190 180 170 160 150 140 130 120 110 100 90 80 70 60 50 40 30 20 10 0
f1 (ppm)

5a11

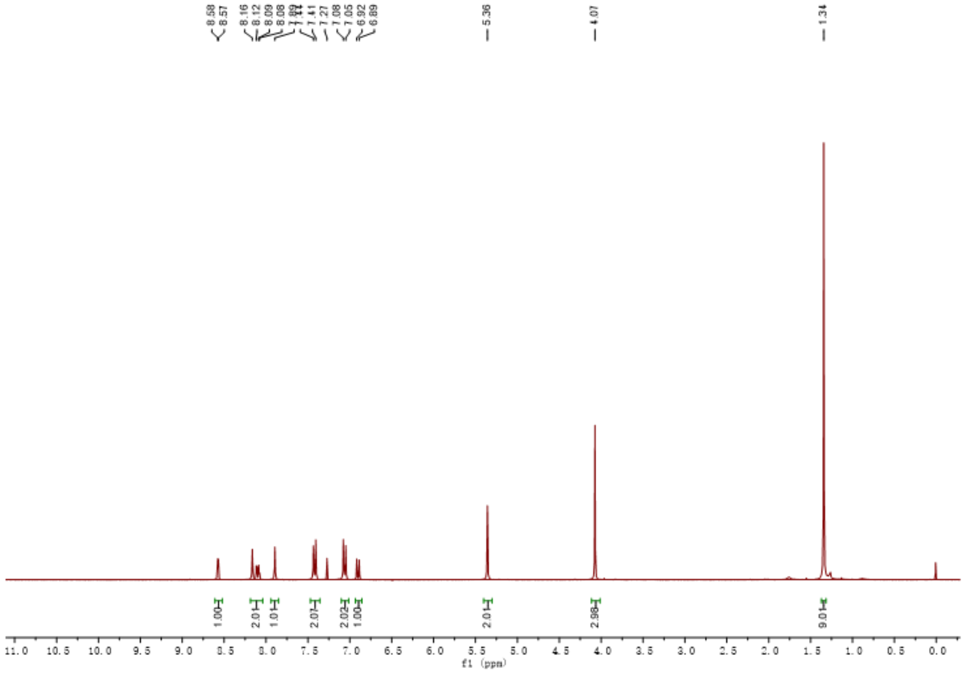
8.58
8.57
8.16
8.12
8.09
8.08
7.41
7.27
7.08
7.05
6.92
6.89
5.36
4.07
1.34
1.00
2.01
1.01
2.07
2.02
1.00
2.01
2.98
9.01
11.0 10.5 10.0 9.5 9.0 8.5 8.0 7.5 7.0 6.5 6.0 5.5 5.0 4.5 4.0 3.5 3.0 2.5 2.0 1.5 1.0 0.5 0.0
f1 (ppm)

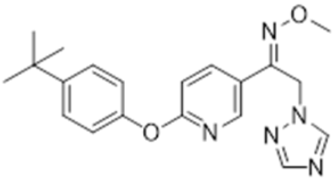

N
O
N
N
N
N

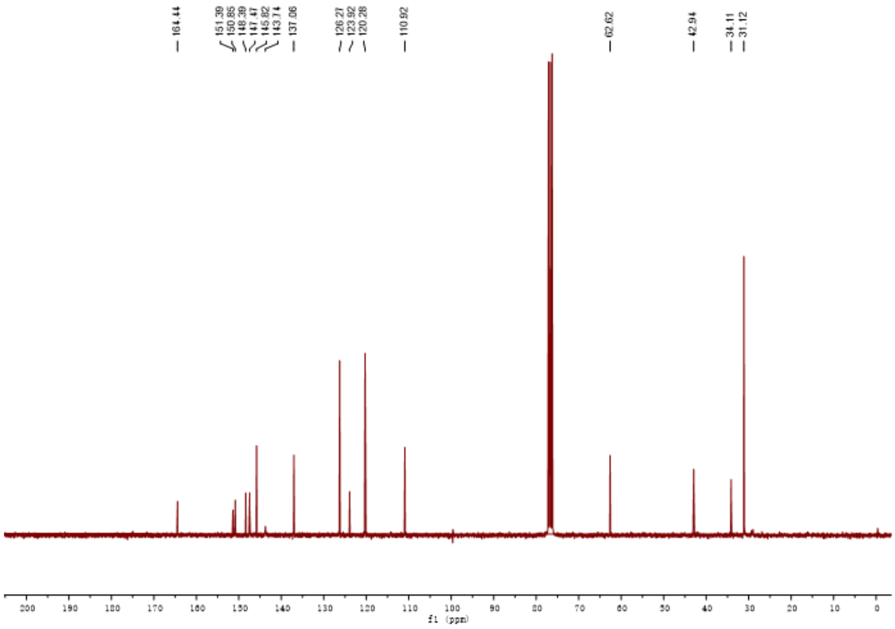

164.44
151.39
150.85
148.39
147.47
145.82
143.74
137.06
126.27
123.92
120.28
110.92
62.62
42.94
34.11
31.12
f1 (ppm)

5a12

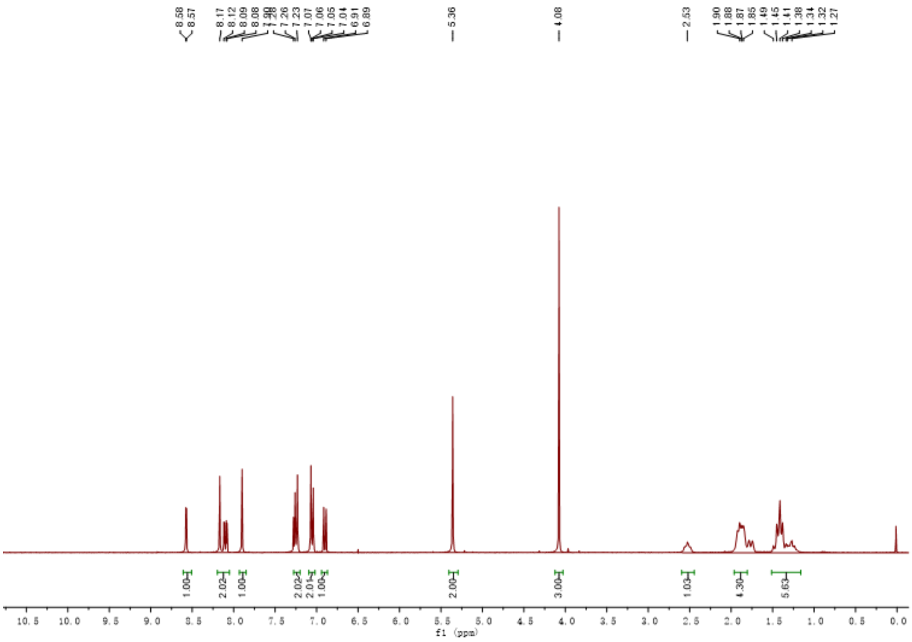

8.58
8.57
8.17
8.12
8.09
8.08
7.26
7.23
7.07
7.06
7.05
7.04
6.91
6.89
5.36
4.08
2.53
1.90
1.88
1.87
1.85
1.49
1.45
1.41
1.38
1.34
1.32
1.27
1.00
2.02
1.00
2.02
2.01
1.00
2.00
3.00
1.03
4.30
5.63
f1 (ppm)

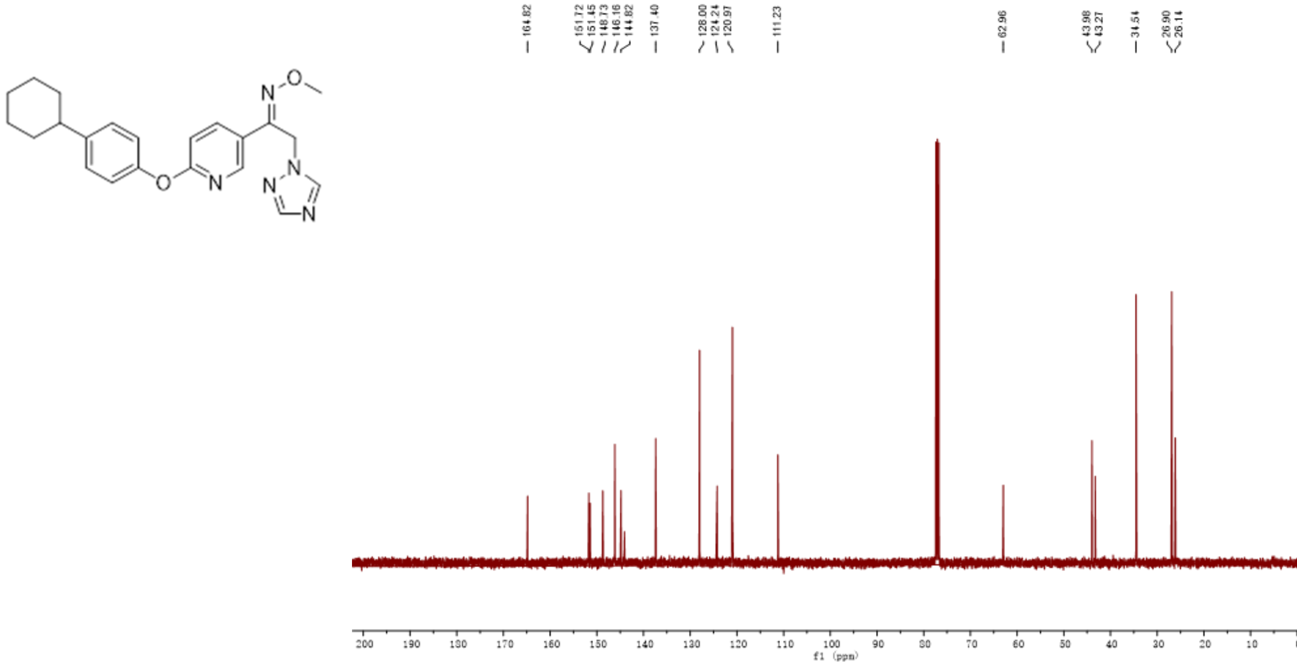
164.82
151.72
151.45
148.73
146.16
144.82
137.40
128.00
124.24
120.97
111.23
62.96
43.98
43.27
34.54
26.90
26.14
f1 (ppm)

5a13

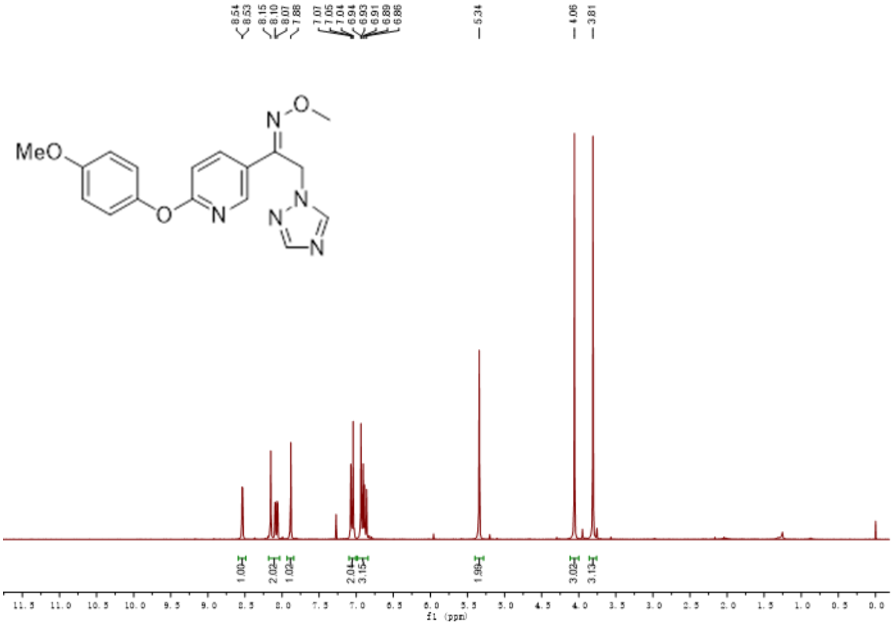
MeO
8.54
8.53
8.15
8.10
8.07
7.88
7.07
7.05
7.04
6.94
6.93
6.91
6.89
6.86
5.34
4.06
3.81
1.00
2.02
1.02
2.04
3.15
1.99
3.02
3.13
f1 (ppm)

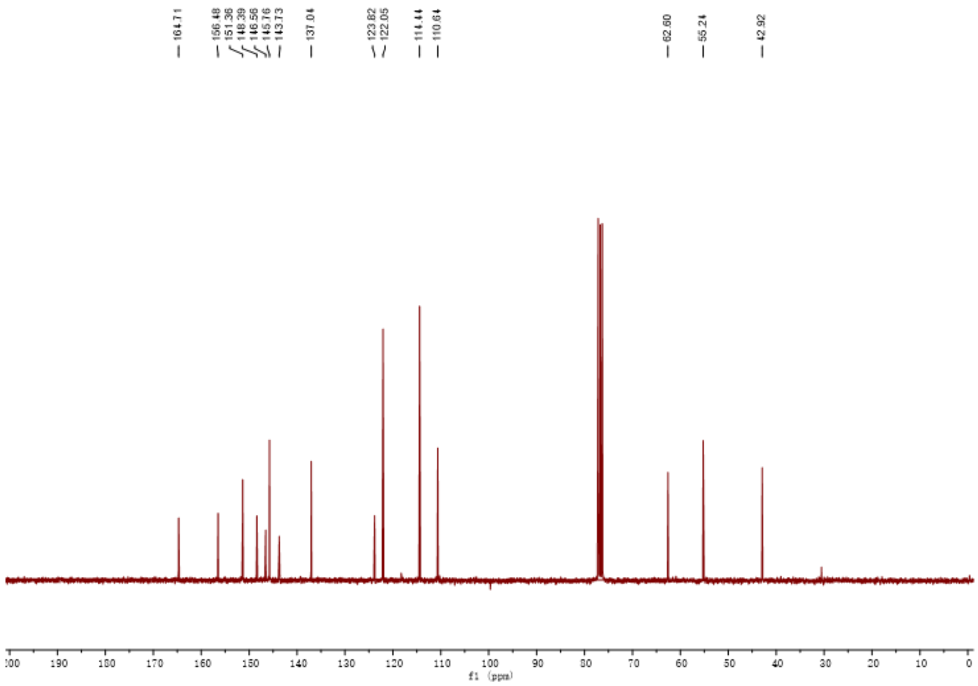
164.71
156.48
151.36
148.39
146.56
145.76
143.73
137.04
123.82
122.05
114.44
110.64
62.60
55.24
42.92
f1 (ppm)

5a14

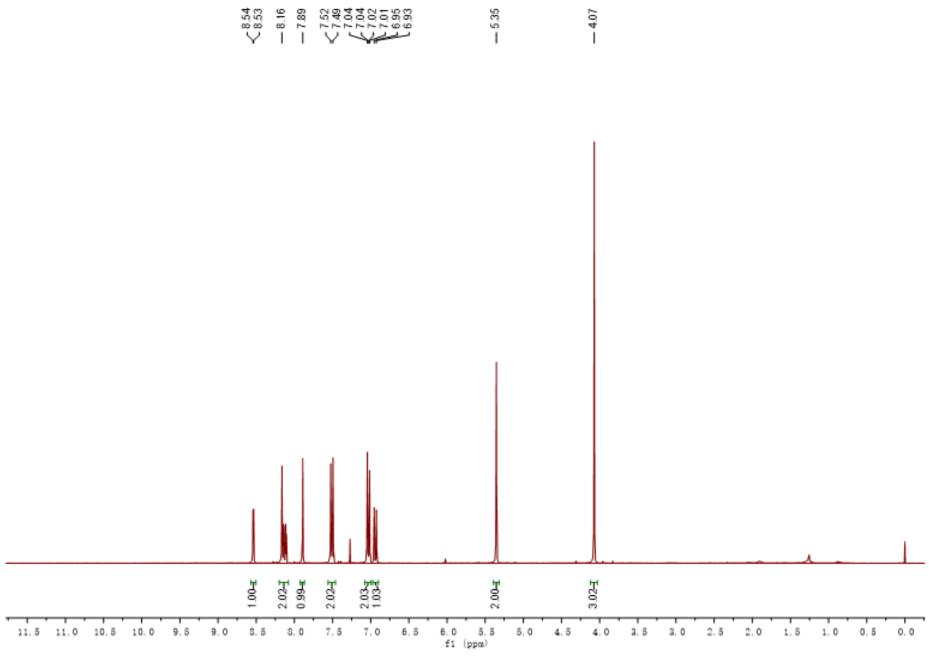
8.54
8.53
8.16
7.89
7.52
7.49
7.04
7.04
7.02
7.01
6.95
6.93
5.35
4.07
1.00
2.02
0.99
2.02
2.03
1.03
2.00
3.02
f1 (ppm)

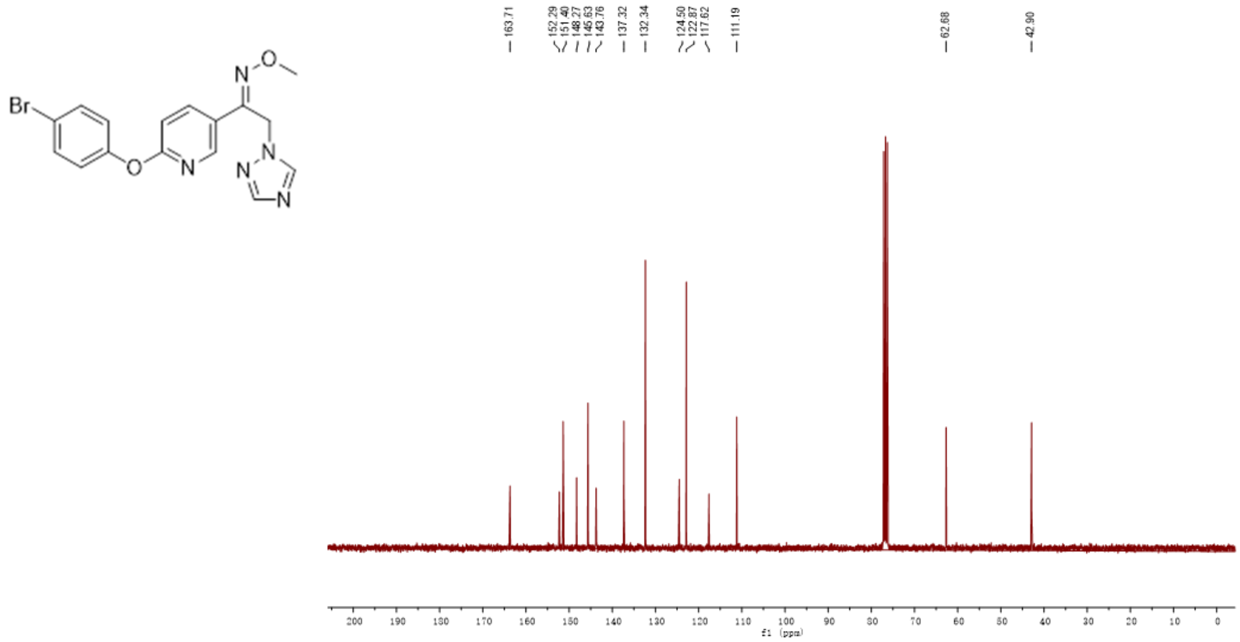

5a15

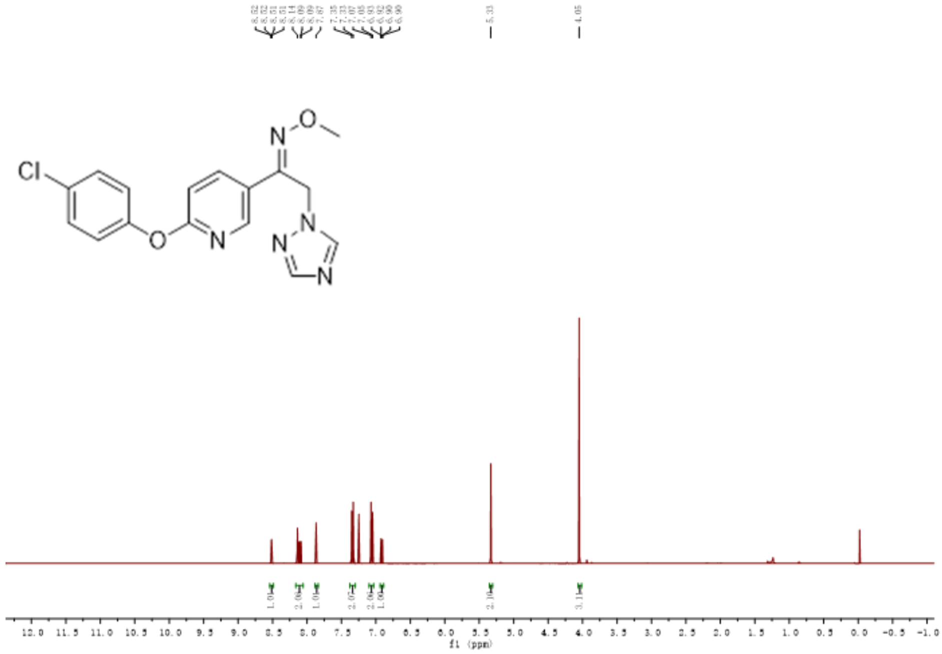

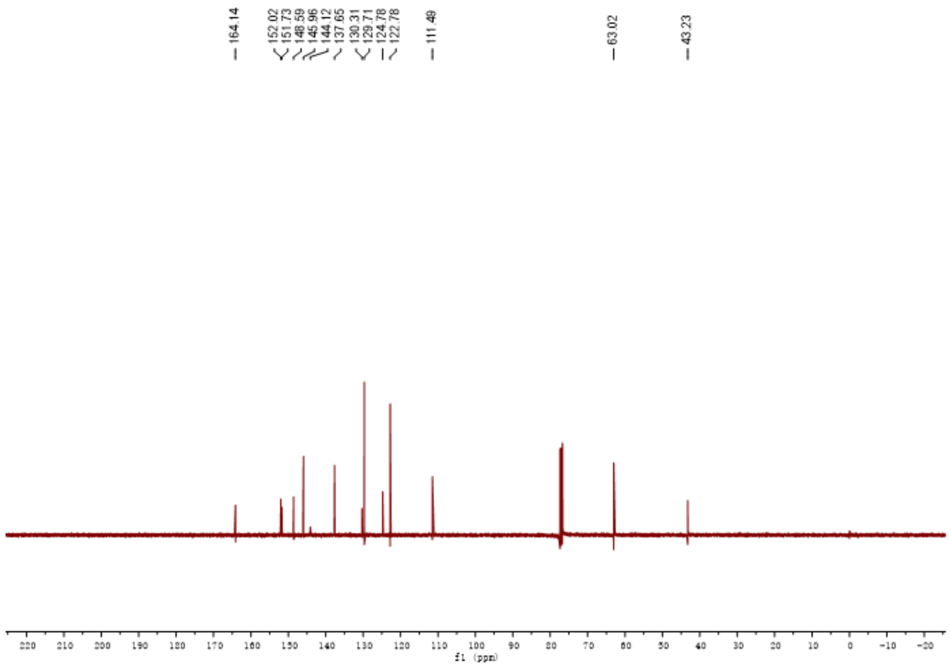

164.14
152.02
151.73
148.59
145.96
144.12
137.65
130.31
129.71
124.78
122.78
111.49
63.02
43.23
f1 (ppm)

5a16

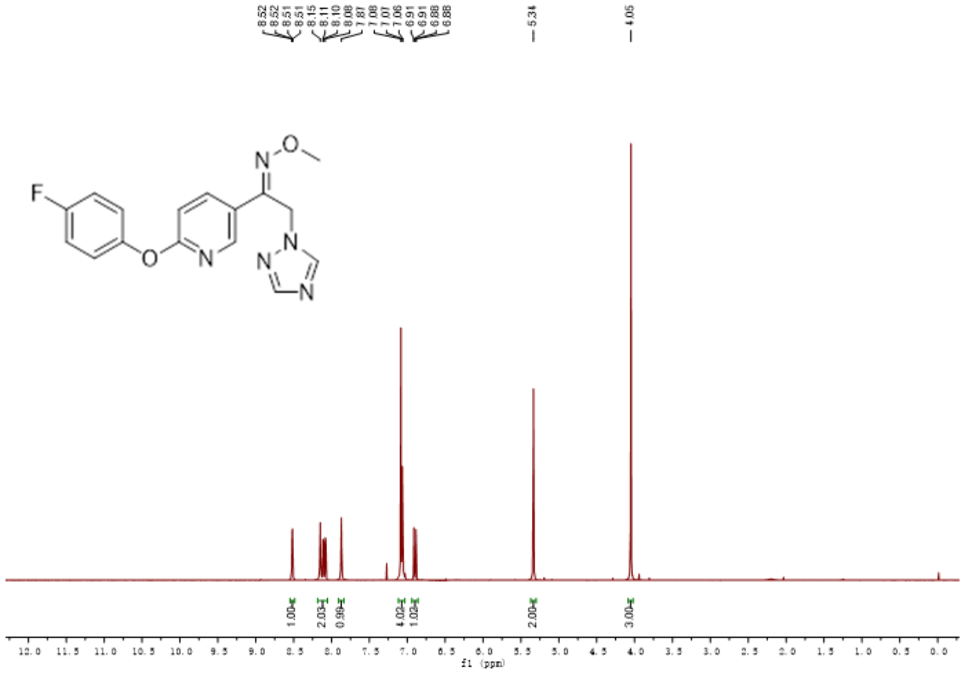

8.52
8.52
8.51
8.51
8.15
8.11
8.10
8.08
7.87
7.08
7.07
7.06
6.91
6.91
6.88
6.88
5.34
4.06
F
O
N
N
O
N
N
N
1.00
2.03
0.99
4.02
1.02
2.00
3.00
f1 (ppm)

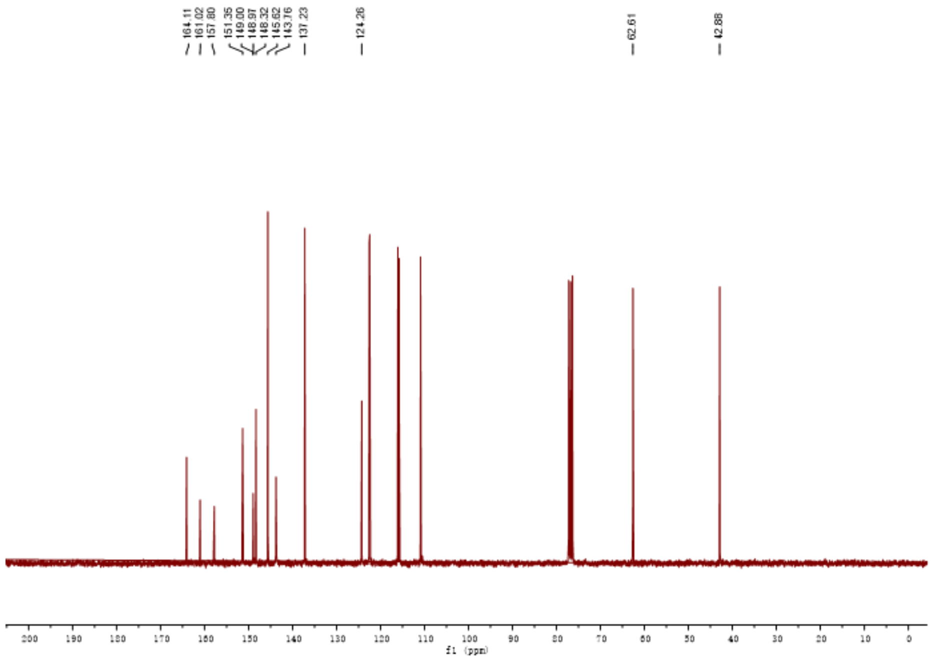
164.11
161.02
157.80
151.35
149.00
148.97
148.32
145.62
143.76
137.23
124.26
62.61
42.88
f1 (ppm)

5a17

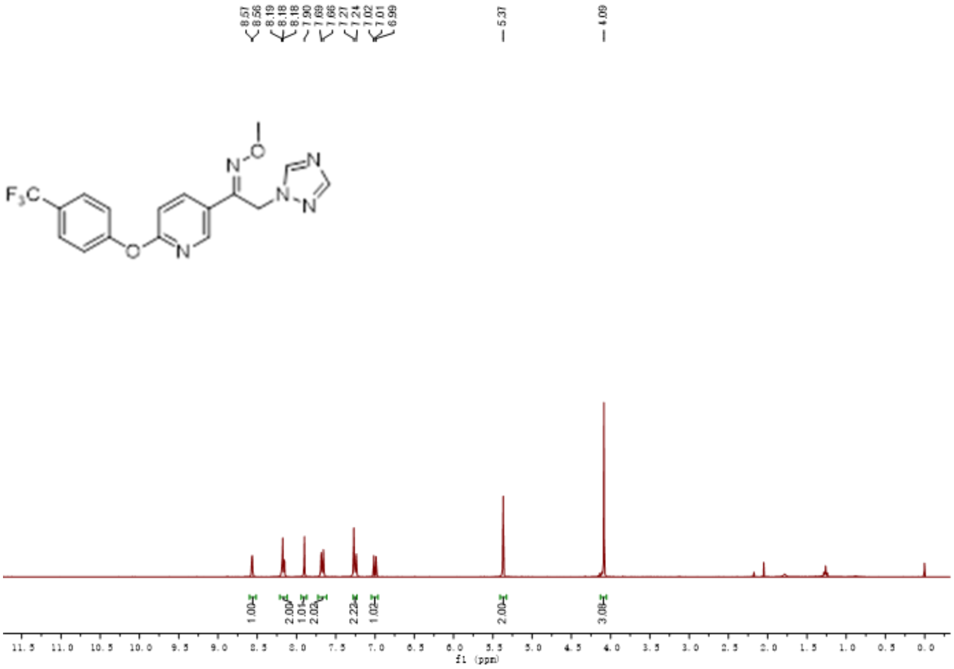
8.57
8.56
8.19
8.18
8.18
7.90
7.69
7.66
7.27
7.24
7.02
7.01
6.99
5.37
4.09
F3C
1.00
2.00
1.01
2.02
2.22
1.02
2.00
3.08
f1 (ppm)

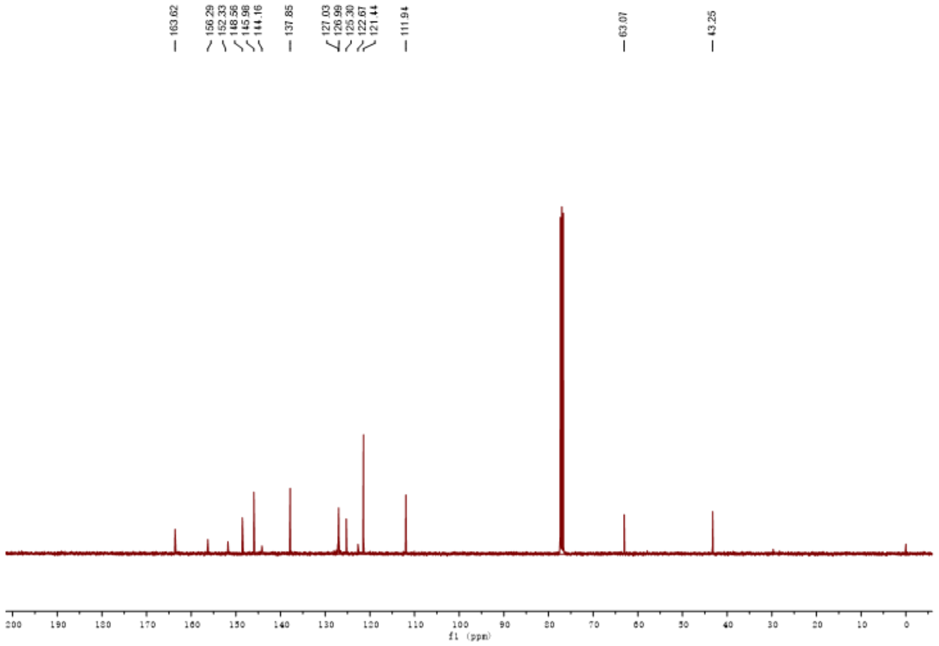
163.62
156.29
152.33
148.56
145.98
144.16
137.85
127.03
126.99
125.30
122.67
121.44
111.94
63.07
43.25
f1 (ppm)

5a19

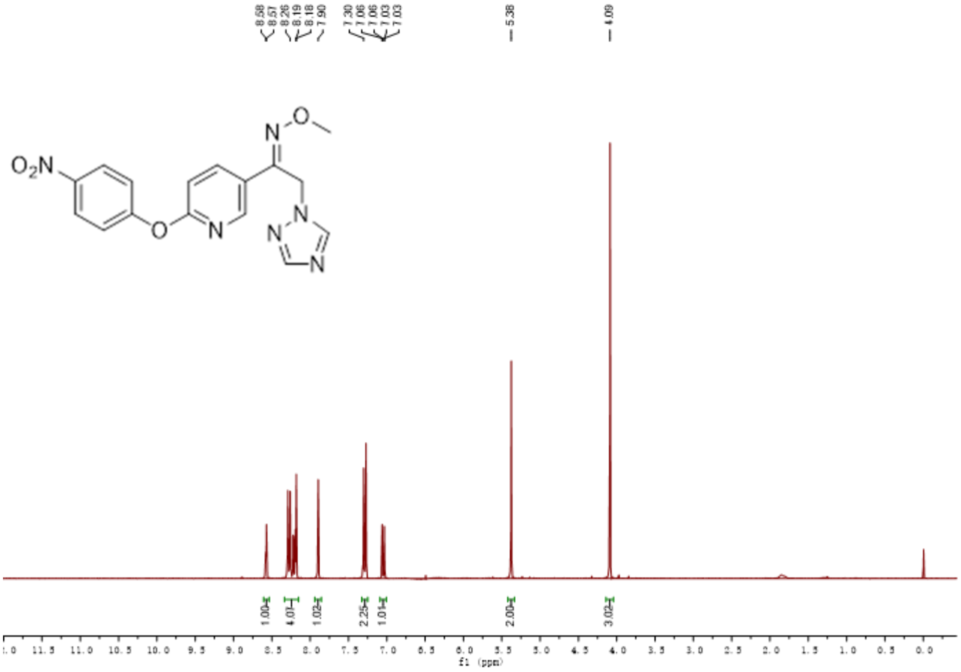
8.68
8.67
8.26
8.19
8.18
7.90
7.30
7.06
7.06
7.03
7.03
5.38
4.09
O2N
1.00
4.01
1.02
2.25
1.01
2.00
3.02
f1 (ppm)

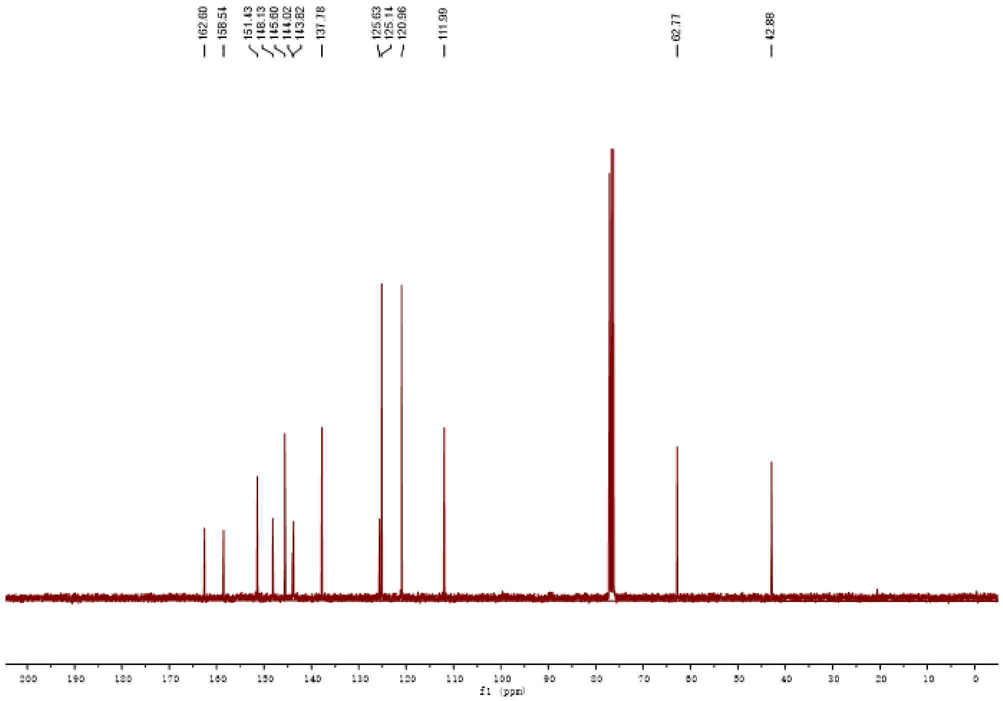
162.60
158.54
151.43
148.13
145.60
144.02
143.62
137.78
125.63
125.14
120.96
111.99
62.77
42.88
f1 (ppm)

5b1

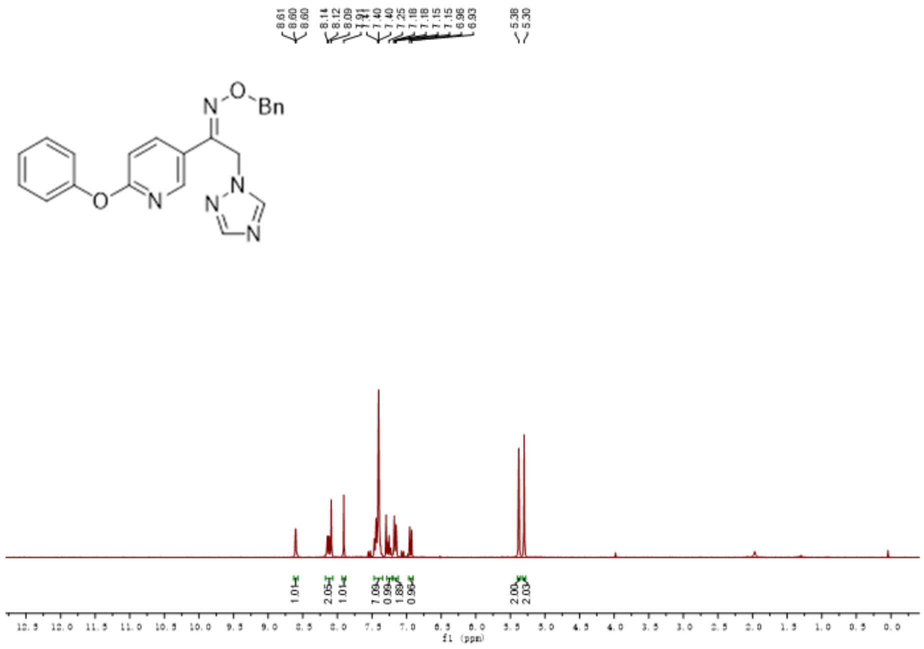
8.61
8.60
8.60
8.14
8.12
6.96
6.93
5.38
5.30
N
O
Bn
N
N
N
N
O
f1 (ppm)

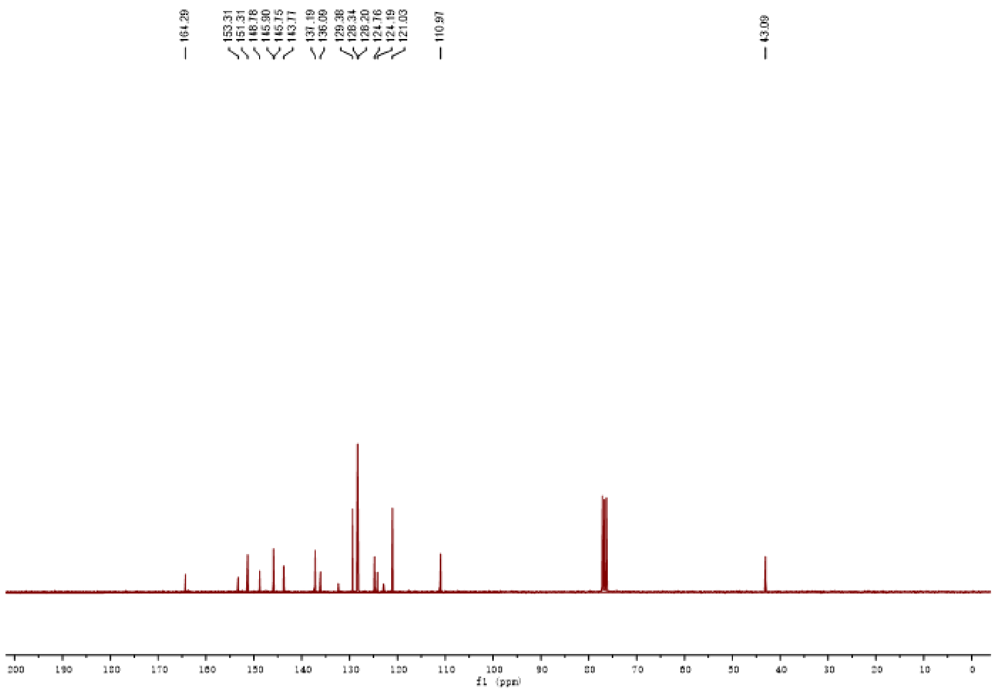

5b2

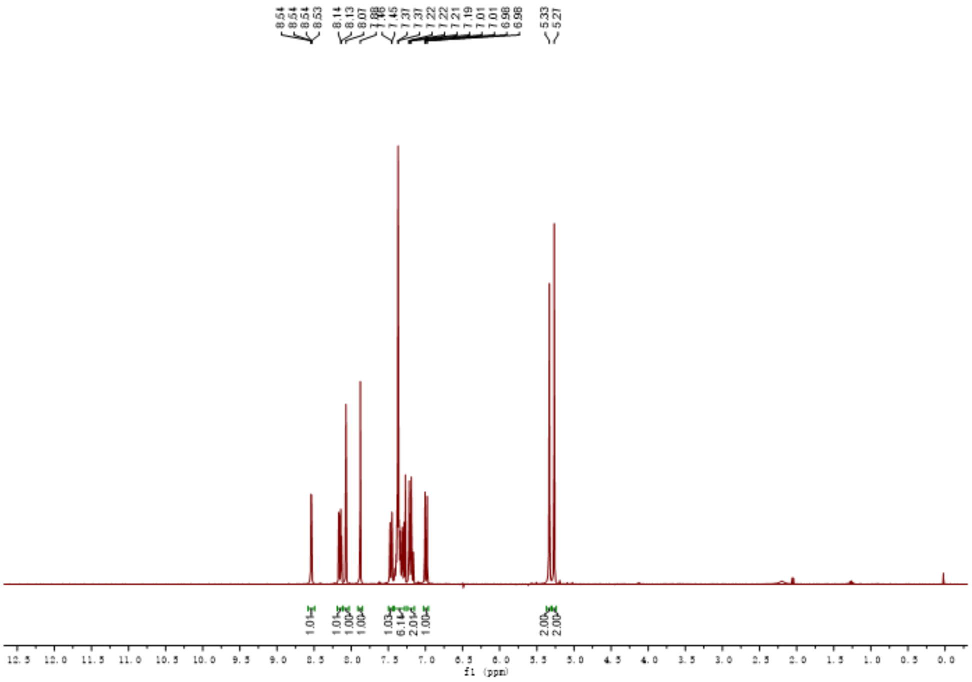

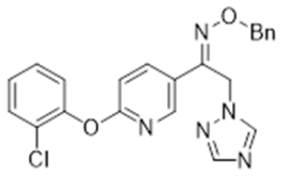

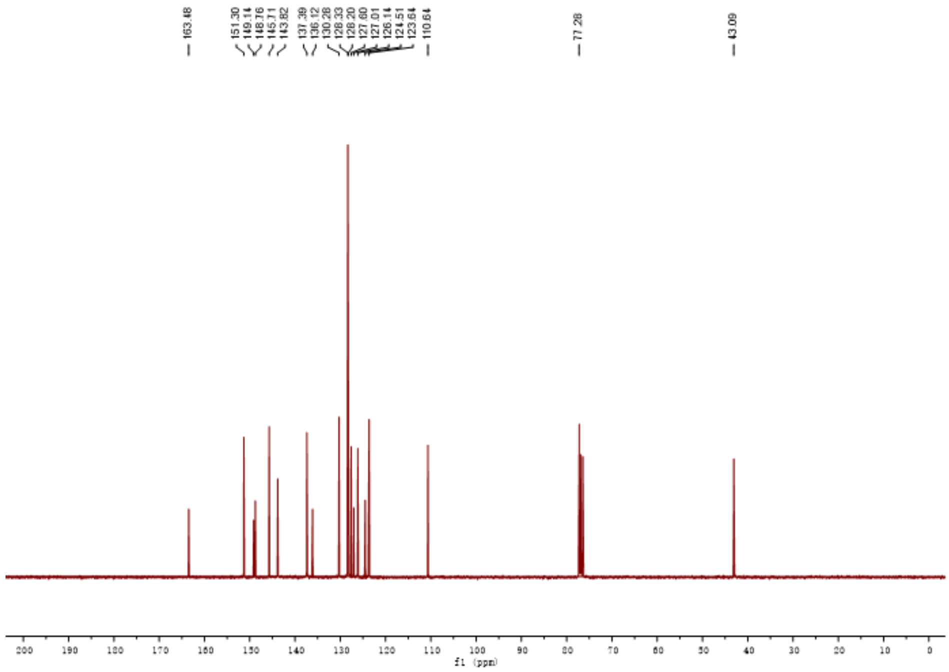

5b3

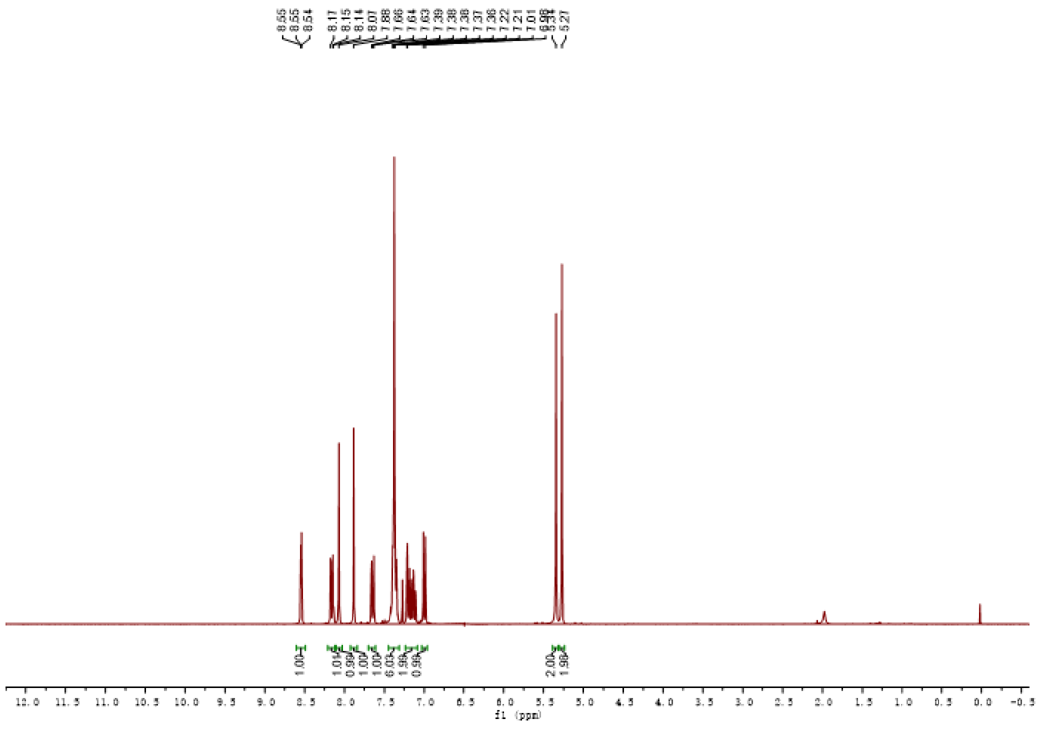

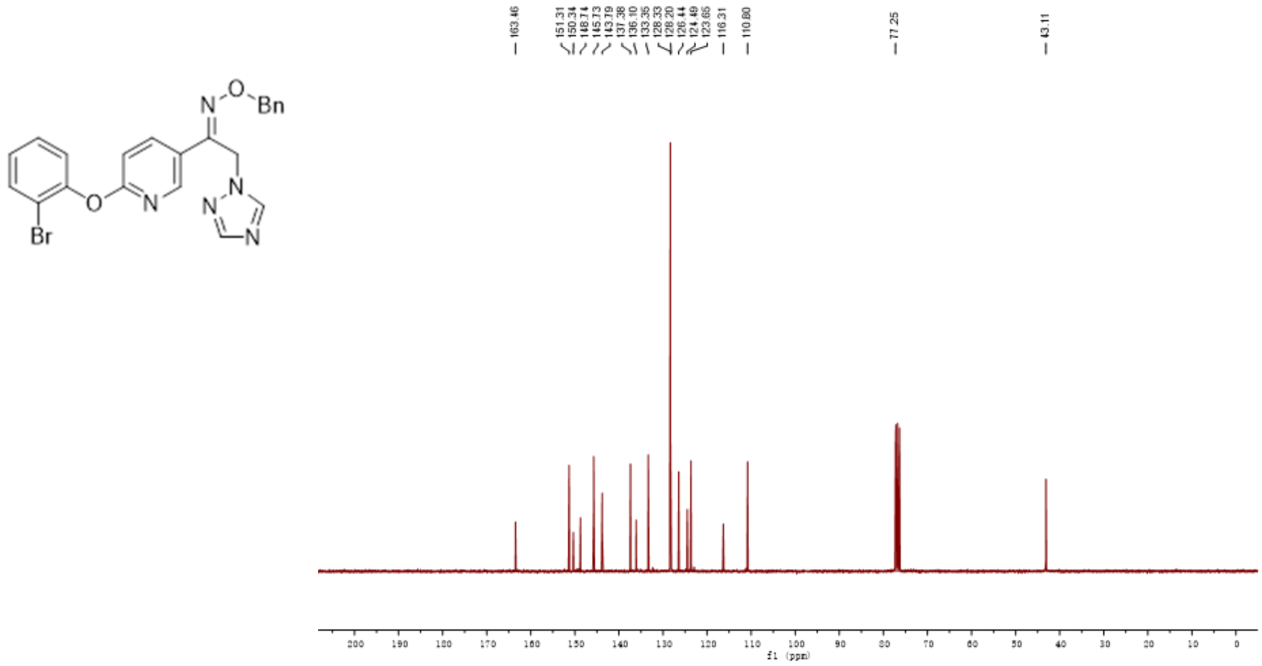

5b4

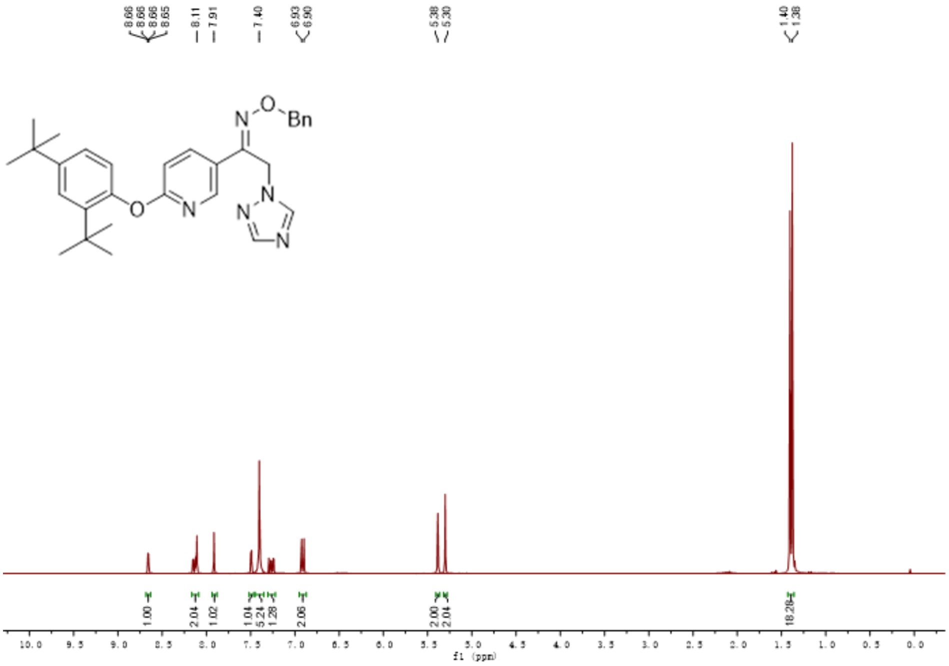

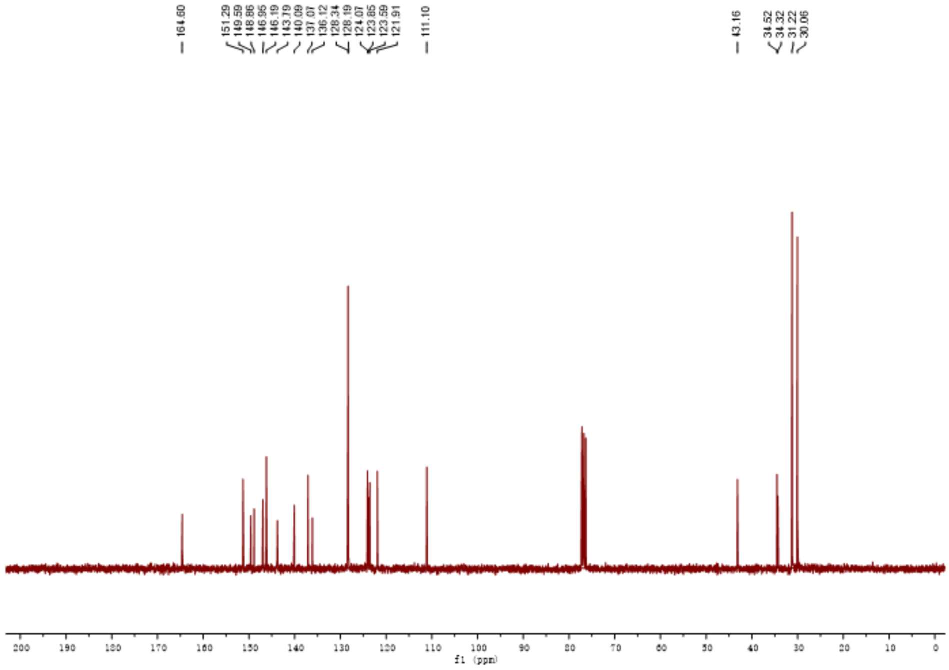
164.60
151.29 149.59 148.86 146.95 146.19 143.79 140.09 137.07 136.12 128.34 128.19 124.07 123.85 123.59 121.91
111.10
43.16
34.52 34.32 31.22 30.06
f1 (ppm)

5b5

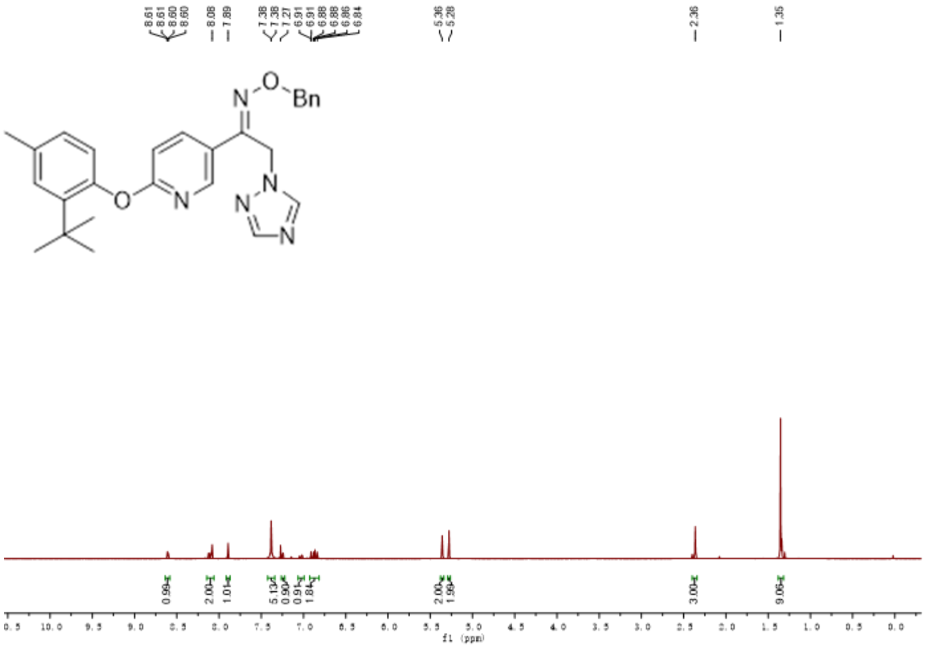
8.61 8.61 8.60 8.60
8.08
7.89
7.38 7.38 7.27 6.91 6.91 6.88 6.88 6.86 6.84
5.36 5.28
2.36
1.35
N O Bn
O N N N N
0.99 2.00 1.01 5.13 0.90 0.91 1.84 2.00 1.99 3.00 9.06
f1 (ppm)

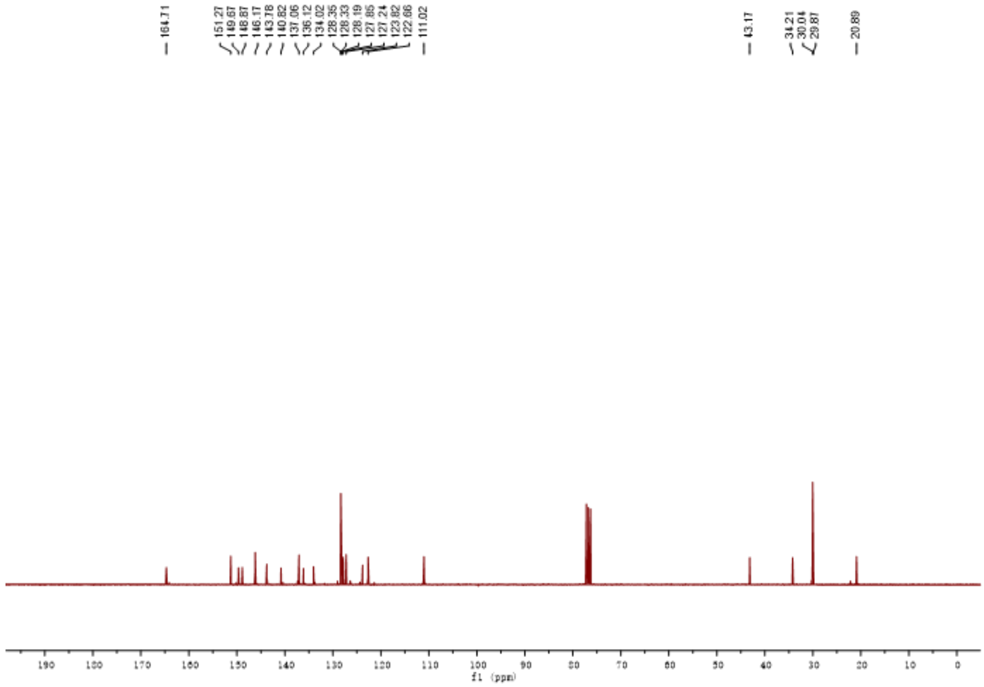
164.71
151.27
149.67
148.87
146.17
143.78
140.82
137.06
136.12
134.02
128.35
128.33
128.19
127.85
127.24
123.82
122.66
111.02
43.17
34.21
30.04
29.87
20.89
f1 (ppm)

5b6

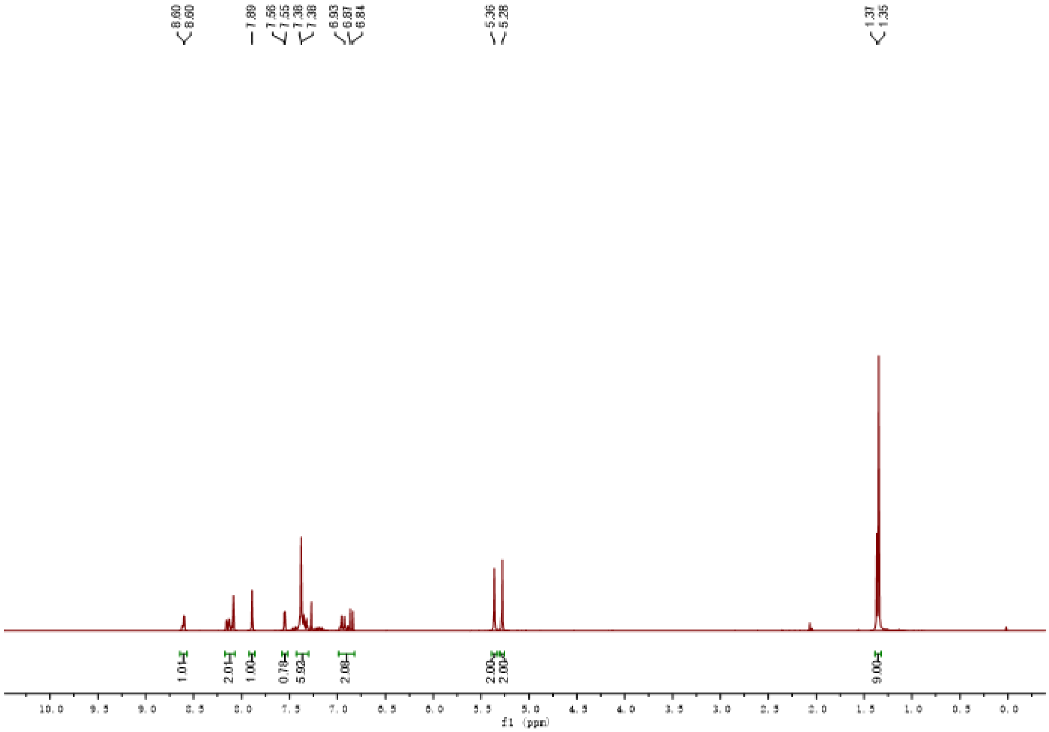
8.60
8.60
7.89
7.56
7.55
7.38
7.38
6.93
6.87
6.84
5.36
5.28
1.37
1.35
1.01
2.01
1.00
0.78
5.92
2.08
2.00
2.00
9.00
f1 (ppm)

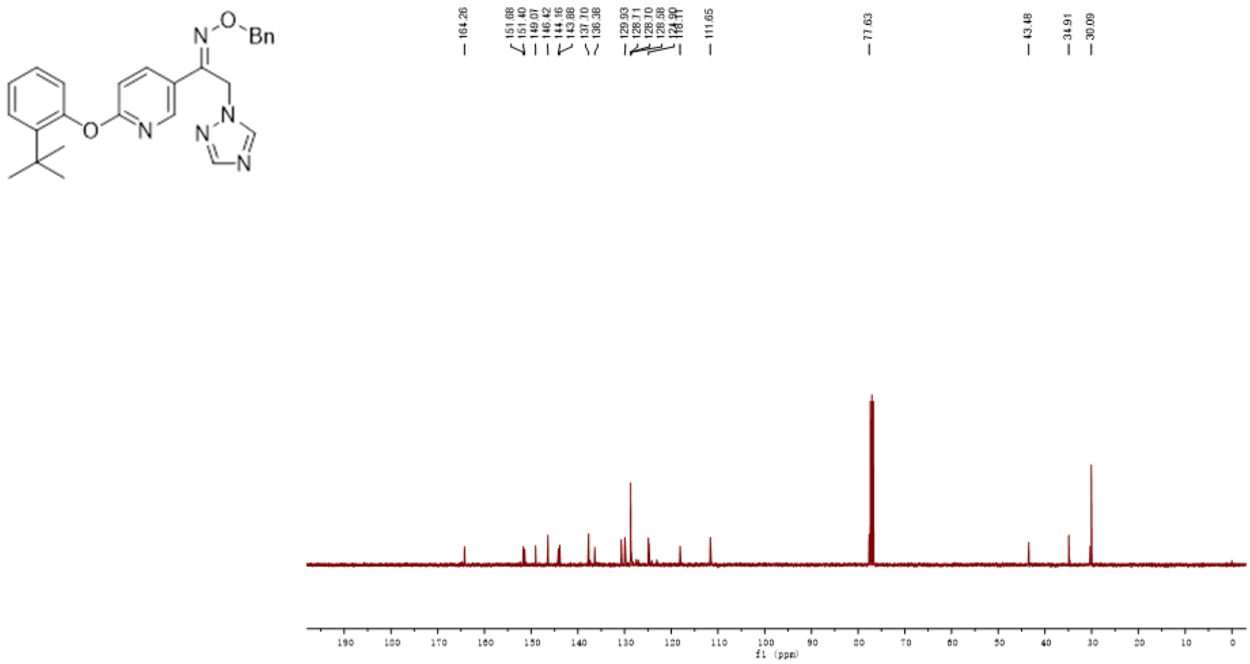

5b7

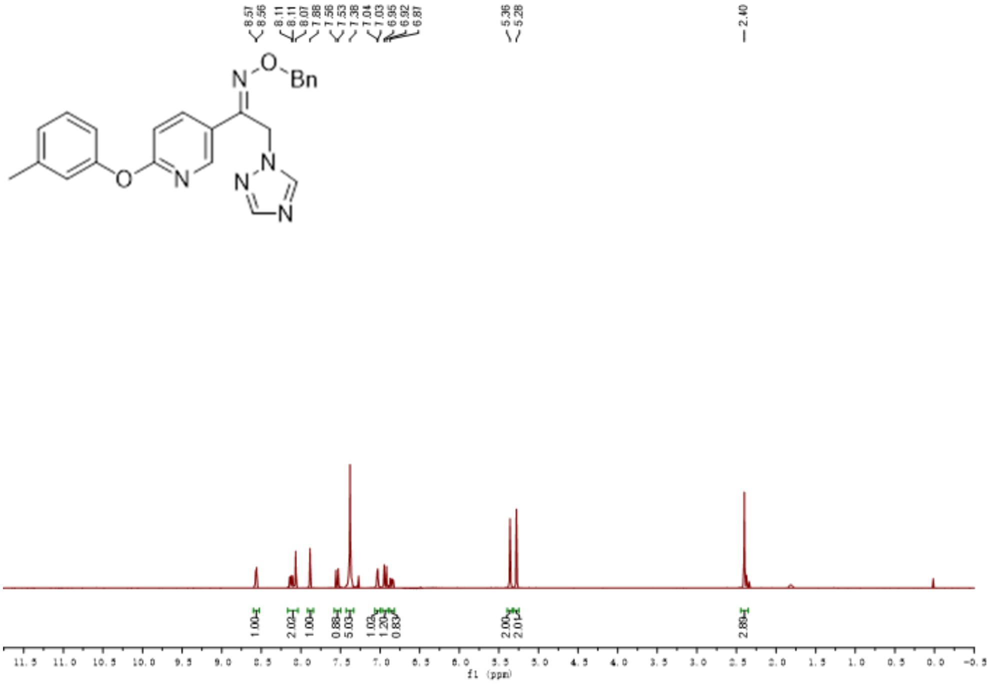

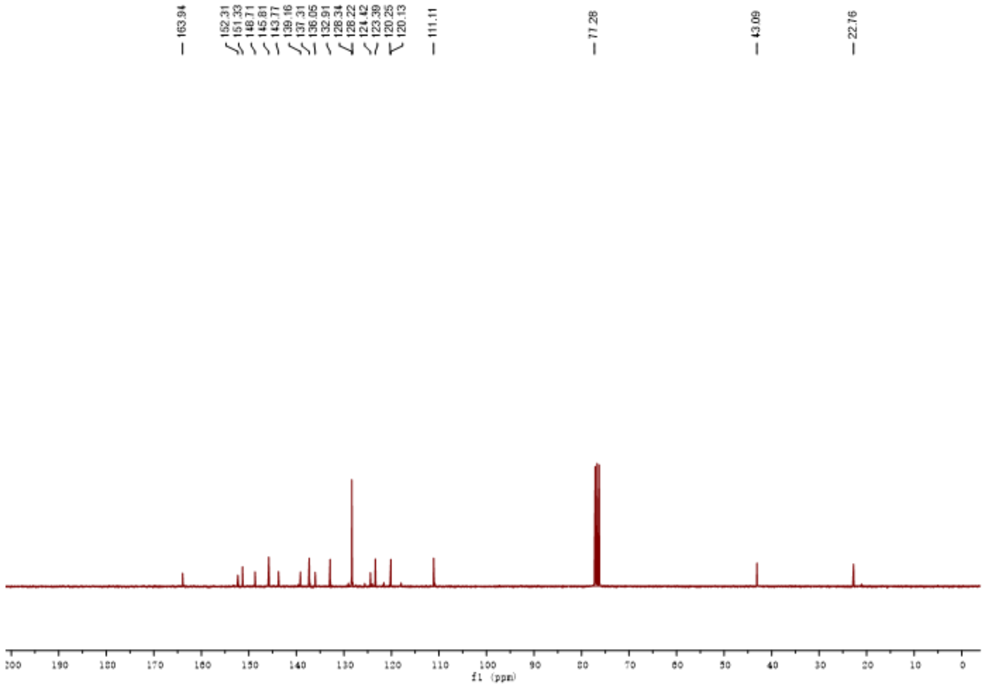
163.94
152.31
151.33
148.71
145.81
143.77
139.16
137.31
136.05
132.91
128.34
128.22
124.42
123.39
120.25
120.13
111.11
77.28
43.09
22.76
f1 (ppm)

5b8

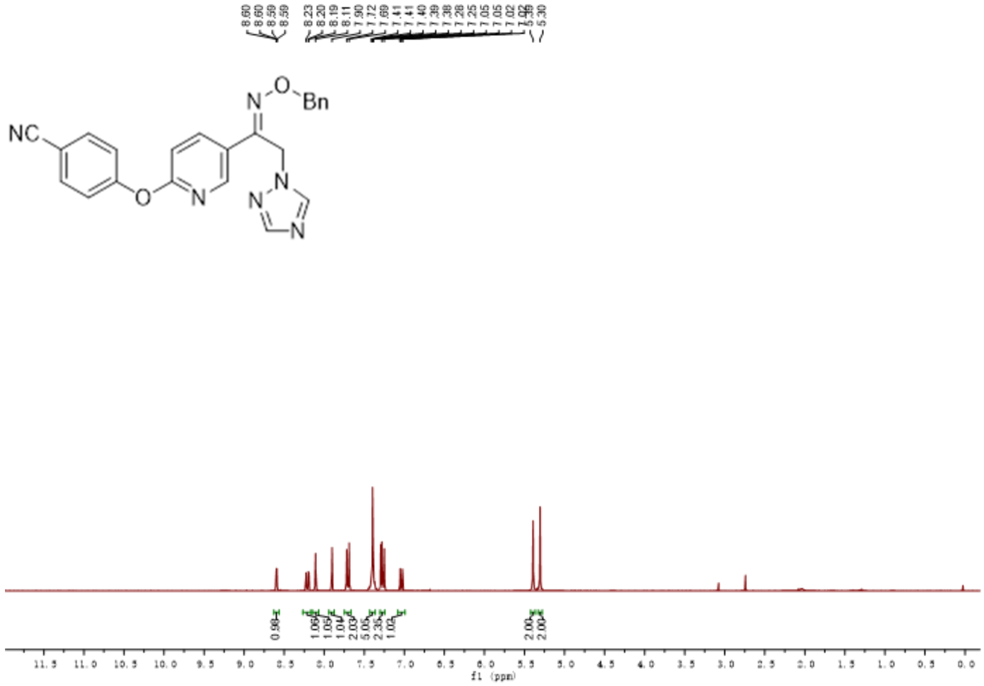
NC
O
N
Bn
f1 (ppm)

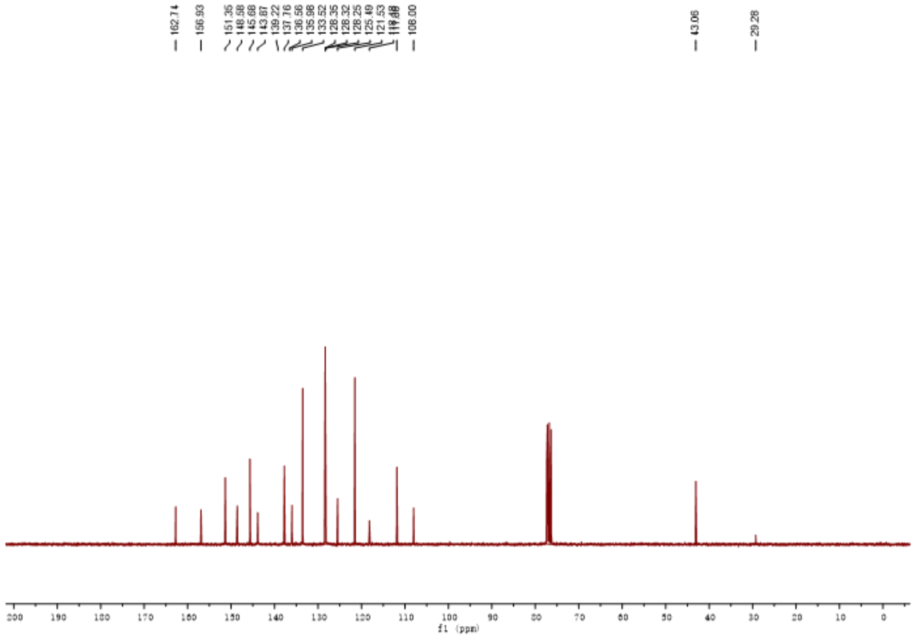
162.74
156.93
151.35
148.58
145.68
143.87
139.22
137.76
136.56
135.98
133.52
128.35
128.32
128.25
125.49
121.53
108.00
43.06
29.28
f1 (ppm)

5b9

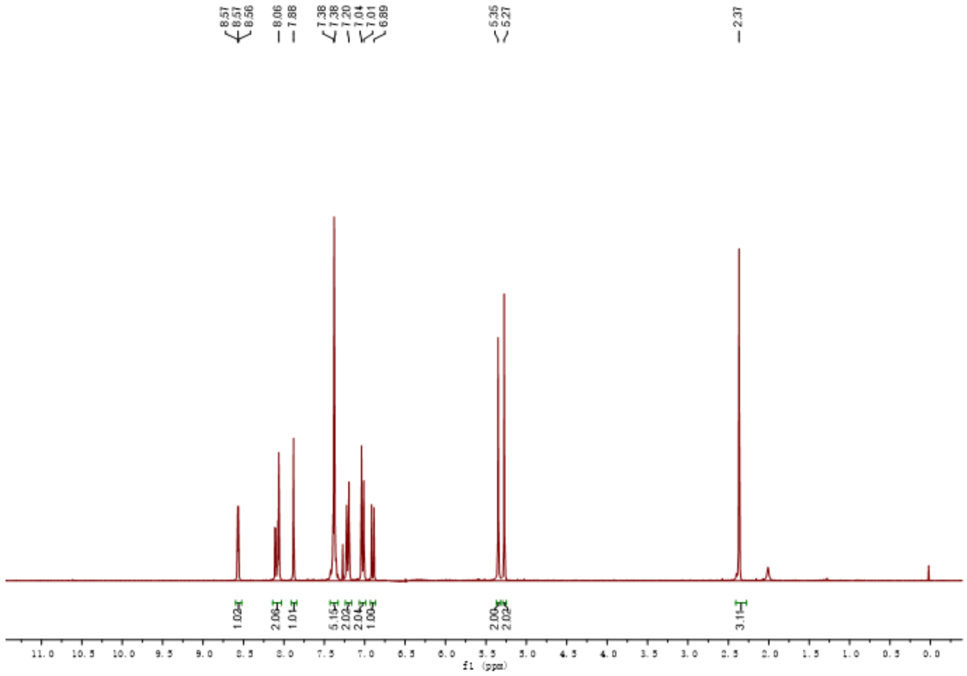
8.57
8.57
8.56
8.06
7.88
7.38
7.38
7.20
7.04
7.01
6.89
5.35
5.27
2.37
1.02
2.06
1.01
5.15
2.02
2.04
1.00
2.00
2.02
3.11
f1 (ppm)

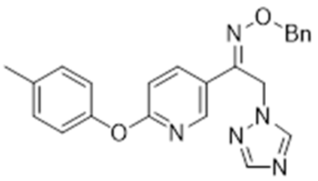
N
O
Bn
O
N
N
N
N

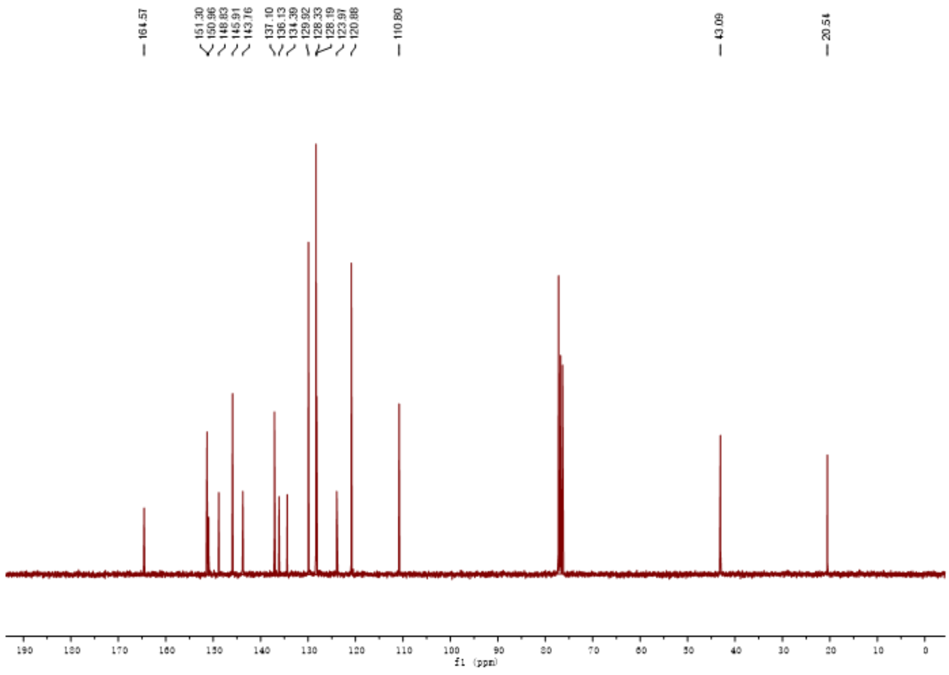
164.57
151.30
150.96
148.83
145.91
143.76
137.10
136.13
134.39
129.92
128.33
128.19
123.97
120.88
110.80
43.09
20.54
190 180 170 160 150 140 130 120 110 100 90 80 70 60 50 40 30 20 10 0
f1 (ppm)
